# Cross-paradigm audit of large language model judges for clinical text evaluation

**DOI:** 10.1038/s41746-026-03443-2

**Published:** 2026-11-04

**Authors:** Tabea M. G. Pakull, Sina Warmer, Kamyar Arzideh, Thomas Hilser, Bernadette Hosters, Hendrik Damm, Felix Nensa, Martin Schuler, Katarzyna Borys, Peter A. Horn, René Hosch, Christoph M. Friedrich

**Affiliations:** 1 Institute for Transfusion Medicine, University Hospital Essen, Essen, Germany; 2 Department of Computer Science, University of Applied Sciences and Arts Dortmund (FHDO), Dortmund, Germany; 3 Institute for Artificial Intelligence in Medicine (IKIM), University Hospital Essen, Essen, Germany; 4 Institute of Diagnostic and Interventional Radiology and Neuroradiology, University Hospital Essen, Essen, Germany; 5 Central IT Department, Data Integration Center, University Hospital Essen, Essen, Germany; 6 West German Cancer Center (WTZ), University Hospital Essen, Essen, Germany; 7 Department of Medical Oncology, University Hospital Essen, Essen, Germany; 8 Department Nursing Development and Nursing Research, University Hospital Essen, Essen, Germany; 9 Institute for Medical Informatics, Biometry and Epidemiology (IMIBE), University Hospital Essen, Essen, Germany; 10 National Center for Tumor Diseases (NCT), NCT West, Essen, Germany

**Keywords:** Computational biology and bioinformatics, Health care, Mathematics and computing, Medical research

## Abstract

Large language models are increasingly being used to evaluate clinical texts, a concept known as LLM-as-a-judge. However, most of the existing evidence focuses on English data and uses pooled agreement metrics that obscure clinically relevant failure modes. This study audited six open-weight models against expert judges on four German clinical datasets covering report and nursing documentation summarization, discharge summary generation, and patient letter generation. The models were evaluated across three paradigms and four prompt configurations. Alignment with experts varied by criterion and corpus, and no configuration proved reliable across paradigms. All judges exhibited position and identity bias. Substituting LLM-as-a-judge for the expert panel shifted 57.2% of pointwise categories, swapped 41.7% of pairwise winners, missed 10.2% of clinical errors, and inverted clinical priorities by overrating the criteria succinct and accurate. Therefore, LLM-as-a-judge is not a safe substitute for experts in German clinical text evaluations, and deployment thresholds should follow decision-impact analysis for LLM-as-a-judge in clinical contexts.

## Introduction

Large language models (LLMs) have become a major focus in medical research. Researchers and clinicians are increasingly exploring LLMs for clinical documentation, patient communication, and decision support^1,2^. As these systems advance toward clinical deployment, the question of whether the generated outputs meet expert standards recurs across application areas. Expert review remains the reference standard but does not scale to the volume of outputs produced during model development and benchmarking, thus motivating the search for automated evaluation methods.

LLM-as-a-judge, a practice in which one LLM evaluates the outputs of another^3^, offers one such method and promises scalable assessment at a fraction of the cost of expert review^4,5^. Research has applied it to score clinical summaries^6^ and reasoning capabilities^7^, and recent large-scale medical benchmarks, such as MedHELM^8^, rely on automated judge scores to rank models across clinical tasks. Clinicians, developers, and hospital governance bodies that select models for downstream use may consult these automated scores. The validity of these decisions, therefore, depends on whether the judge agrees with experts under the conditions of intended use. However, this agreement may vary by task, model, prompt, or evaluation criteria.

Current practice spans different judgment formats^4,9^ that carry distinct failure modes. The pointwise paradigm underlies rubric frameworks such as the Provider Documentation Summarization Quality Instrument (PDSQI-9)^10^ and requires judges to assign a categorical score per criterion. Failures can lead to over- or underestimates of quality, which may cause unfit models being selected for deployment. In the pairwise paradigm, the judge receives two candidate texts and selects one under a comparative criterion. This paradigm dominates arena-style benchmarks^3^. Failures can misdirect model selection decisions. The pass-or-fail paradigm requires judges to flag whether a defined violation is present in the text. This format is closest to a safety guardrail. Failures allow either harmful content to pass through or false alarms to be generated, both of which erode clinician trust.

The evidence that supports LLM-as-a-judge in clinical contexts comes from English data and rests on pooled agreement statistics computed over pooled samples. Reported figures include “almost perfect agreement” (0.818) against human raters at a median score difference of zero for PDSQI-9 scoring of English electronic health record summaries^6^, “moderate agreement” (0.47) of an LLM jury against clinician ratings above the clinician-clinician value^8^, and 93.2% agreement with clinicians above the highest clinician-clinician agreement of 88.5% for statement-level fact verification^11^. However, pooled statistics can hide failure modes per criterion and in cases with different annotation consensus^12^. Evaluations of LLM-as-a-judge in the general domain also raise measurement-theoretic critique of construct validity and reliability^13^, preference for stylistic fluency over factual correctness^14^, and position bias in pairwise comparisons^15^. Further, LLM-as-a-judge consistency drops for non-English text^16^. German clinical LLM work targets text generation rather than judge evaluation^17^.

This study audits six open-weight LLMs as judges across the described three paradigms, using a simple baseline prompt, a rule-augmented operationalization, a few-shot demonstration, and a multiagent ensemble configuration. It uses four German, expert-annotated datasets drawn from routine documentation at a German academic university hospital that cover core documentation tasks. Clinical report^18^ and nursing documentation^19^ summarization, discharge summary generation^18^, and molecular tumor board patient letters generation^20^. Because LLM judges show closer alignment with laypeople than with experts^21^, all reference labels used in this study come from clinical experts in the relevant specialty. The restriction to open-weight models follows European deployment conditions, under which the General Data Protection Regulation (GDPR) restricts transmission of patient health information to external commercial servers.

The audit reports LLM-as-a-judge performance for German clinical text evaluation beyond inter-rater reliability. It shows alignment with expert consensus per criterion and stratifies it by the level of expert consensus. The failure modes documented outside the clinical domain are tested directly, covering position bias under reversed presentation order, identity bias between human-written and model-generated candidates, and variability across repeated runs. A decision-impact case study then examines whether the substitution of LLM-as-a-judge for the expert panel changes the conclusions clinical AI researchers, benchmark developers, and hospital governance bodies would draw.

## Results

The following sections report judge performance measured by absolute alignment, reliability, and robustness for six open-weight LLMs ranging from 24B to 235B parameters. The case study further exemplifies how substituting the human panel with an LLM-as-a-judge can affect decision-relevant metrics.

### Absolute alignment between judges and experts

**Fig. 1:**
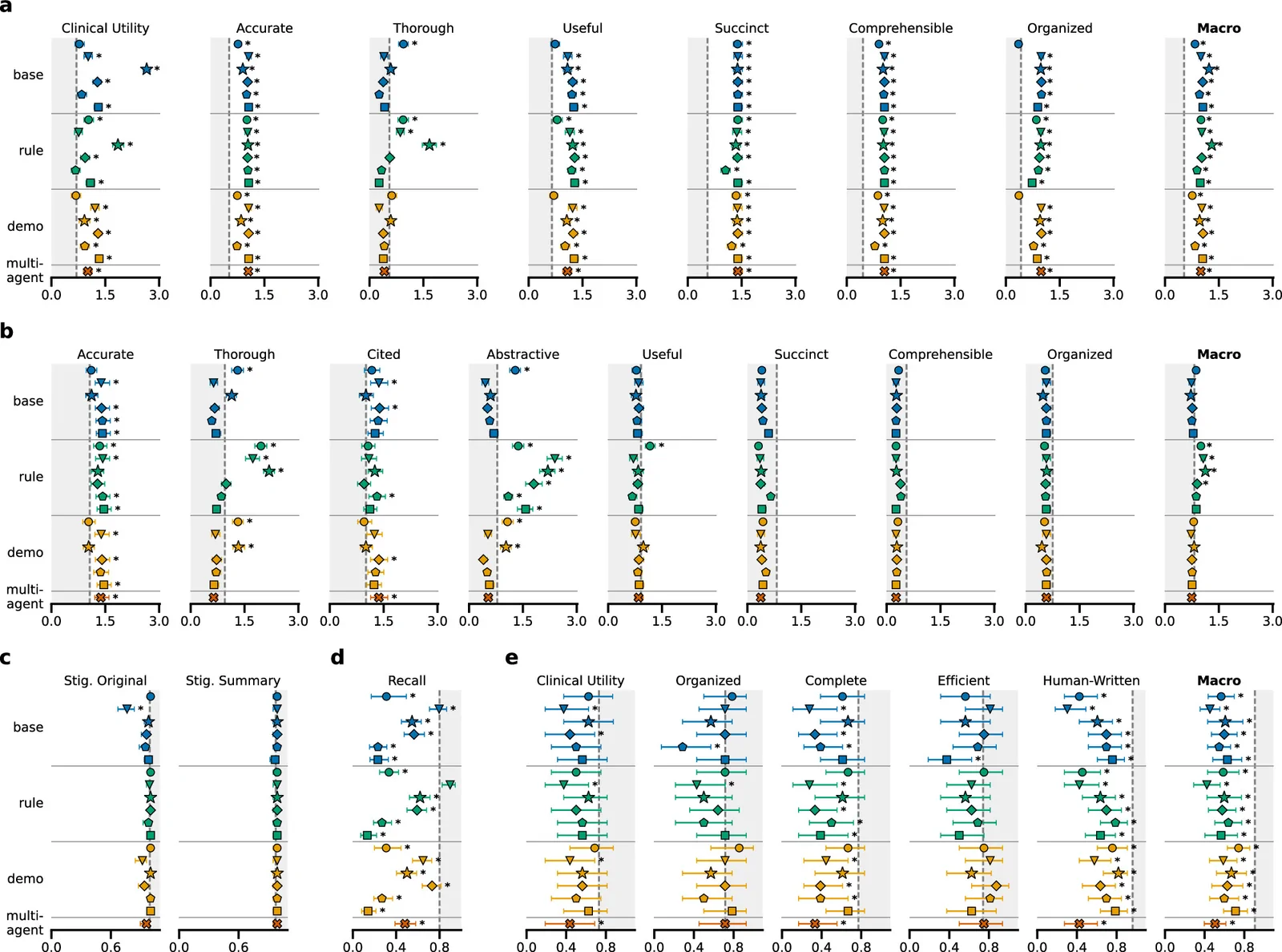
Absolute alignment of LLM judges with experts. Each model-prompt combination is shown as its own row, with point estimate and bootstrap 95% confidence interval in each criterion facet. Rows are grouped by prompt configuration and ordered by model within a group. **a** Reports (pointwise): mean absolute error (MAE) per criterion, plus a pooled Macro facet. **b** Nursing (pointwise): MAE per criterion, plus a pooled Macro facet. **c** Nursing (pointwise): accuracy per binary stigma criteria. **d** MTB (pass-or-fail): recall for clinically harmful content. **e** Discharge summary (pairwise): accuracy per criterion, plus a pooled Macro facet. Point color denotes the prompt configuration (blue: baseline, green: rule, orange: demo, red: multiagent) and marker shape the judge model (circle: gpt-oss-120b, downward triangle: Qwen3-235B-A22B-Instruct-2507-FP8, star: Qwen3-235B-A22B-Thinking-2507-FP8, diamond: Qwen3-Next-80B-A3B-Instruct, pentagon: Magistral-Small-2509, square: medgemma-27b-it, cross: MULTIAGENT). The dashed lines indicate the mean human threshold for mean absolute error (MAE) and accuracy, and the grey band shades the side of that line which is better than the human reference. Asterisks denote model-prompt combinations significantly worse than the threshold. Macro facets pool all items across a dataset’s criteria. Exact numbers can be found in Supplementary Tables 6–9.

Alignment with expert ratings varied substantially by task, criterion and prompt configuration (Fig. 1).

In the pointwise paradigm, alignment was measured as the mean absolute error (MAE) between each judge’s predicted score and the median score of the human experts for every item and ordinal criterion.

On the reports dataset (Fig. 1a), the macro-averaged MAE of all judges was significantly (FDR-corrected *p* < 0.05) worse than the human threshold (0.53). The *succinct* criterion showed the largest, consistent divergence from human judgment across all judges (1.05–1.40).

For some of the criteria, a few of the judges performed at the human level. Notably, gpt-oss-120b/baseline and gpt-oss-120b/demo had MAE that cleared the threshold or were statistically indistinguishable from the human threshold for the *clinical utility*, *useful*, and *organized* criteria. Most judges cleared the threshold for *thorough*, although the total range was wide (0.27–0.94) due to four outliers (Qwen3-235B-A22B-Thinking-2507-FP8/rule 1.67, 95% CI [1.47, 1.86], gpt-oss-120b/baseline 0.94, 95% CI [0.81, 1.07], gpt-oss-120b/rule 0.93, 95% CI [0.79, 1.08], and Qwen3-235B-A22B-Instruct-2507-FP8/rule 0.86, 95% CI [0.75, 0.96]).

On the nursing dataset (Fig. 1b), most judges performed at a level that was statistically indistinguishable from the human threshold of 0.82. Rule-augmented prompting caused some models to perform worse, particularly for the *abstractive* and *thorough* criteria. Consequently, these models exhibited significantly worse macro-MAE than the human threshold. The widest spread of MAE was observed for *abstractive* (0.40–2.39) followed by *thorough* (0.59–2.19).

For some criteria the choice of model and configuration had little to no effect. The criteria *comprehensible* (0.26–0.40), *organized* (0.45–0.58), and *succinct* (0.31–0.65) had similar MAE for all judges that all clear the respective thresholds. For the criteria *stig. original* and stig. *summary* (identifying stigmatizing language in the original and generated texts, Fig. 1c) accuracy instead of MAE was used to measure performance. Most judges achieved an accuracy >0.92, with a single significant outlier for *stig. original* (Qwen3-235B-A22B-Instruct-2507-FP8/baseline).

In the pass-or-fail paradigm (Fig. 1d), in which judges were assigned to detect errors in text sections, the recall ranged from 0.90 (Qwen3-235B-A22B-Instruct-2507-FP8/rule, 95% CI [0.82, 0.95]) to 0.13 (medgemma-27b-it/rule, 95% CI [0.08, 0.22]). The scores show model-specific performance patterns with limited effect of prompt configurations.

In the pairwise paradigm (Fig. 1e), two competing discharge summaries were presented to the judges in both presentation orders. Alignment was measured as accuracy against the strict majority of the expert raters, with 2-2 splits analyzed as ambiguous.

Accuracy varied substantially by criterion, and no judge surpassed the threshold (0.91). Specifically, in the *human-written* criterion all models performed significantly worse than humans. Some accuracy values exceeded the threshold for the *efficient* and *organized* criteria. Overall, the ranges were wide, with some judges having accuracy below chance level. No clear criterion trend was visible, and confidence intervals were wide for all judges.

An exploratory analysis of reasoning capabilities revealed that they did not lead to consistent improvements in alignment with expert consensus. Regarding binary nursing criteria, the reasoning-enabled variant of Qwen3-235B-A22B-Instruct-2507-FP8, Qwen3-235B-A22B-Thinking-2507-FP8, had higher accuracy (FDR-corrected *p* < 0.001 for baseline and *p* = 0.012 for demo). However, this advantage did not extend to ordinal severity ratings. In these ratings, the instruct model had a significantly lower MAE in the reports corpus across all three configurations tested (e.g., 1.02 vs. 1.30 for rule config; FDR-corrected *p* < 0.001) and in two of the three configurations tested for nursing. In the pairwise paradigm, there were no significant differences between Qwen3-235B-A22B-Instruct-2507-FP8 and Qwen3-235B-A22B-Thinking-2507-FP8. In the pass-or-fail error-detection task, the reasoning-enabled model had higher overall precision but significantly lower recall of clinical errors in all three configurations (e.g., 0.62 vs. 0.90 for rule config; FDR-corrected *p* < 0.001).

### Reliability relative to the expert panel

**Fig. 2:**
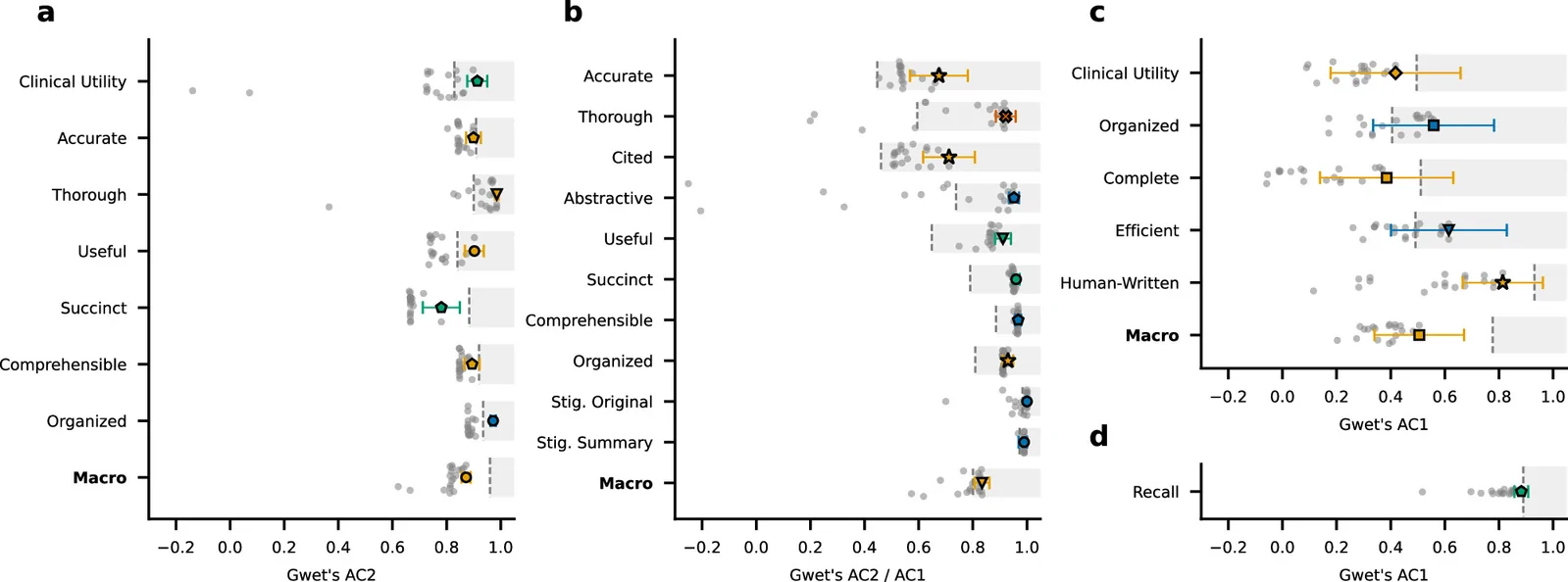
Inter-rater reliability between LLM judges and experts. Reliability expressed as chance-corrected Gwet’s agreement coefficient (AC), one panel per evaluation setting. **a** Reports (pointwise), **b** Nursing (pointwise), **c** Discharge summary (pairwise), **d** MTB (pass/fail). Each panel has one row per criterion plus a pooled Macro row where applicable. Each criterion carries a dashed horizontal reference line at the human-human AC, and the grey band shades the side of that line which is better than the human reference. Marker shape denotes the judge model achieving the highest AC (circle: gpt-oss-120b, downward triangle: Qwen3-235B-A22B-Instruct-2507-FP8, star: Qwen3-235B-A22B-Thinking-2507-FP8, diamond: Qwen3-Next-80B-A3B-Instruct, pentagon: Magistral-Small-2509, square: medgemma-27b-it, cross: MULTIAGENT) and its fill color the prompt configuration (blue: baseline, green: rule, orange: demo, red: multiagent). The small grey jittered points behind each row show the full spread, one per model-prompt combination. Macro rows pool all items across a dataset’s criteria.

Reliability of the judges against the human inter-rater reliability is shown in Fig. 2.

Reliability was defined as the chance-corrected agreement between each LLM judge and the human consensus rating (Gwet’s AC2^22^ with quadratic weights for ordinal criteria, Gwet’s AC1^22^ for binary and pairwise criteria). Because chance-corrected agreement is not comparable across criteria of differing difficulty, every judge was interpreted against the human–human agreement computed on the same items. On the Landis and Koch scale, the human inter-rater reliability was “almost perfect” for the reports dataset and MTB dataset (Figs. 2a, d) and “substantial” for the discharge summary dataset (Fig. 2c).

Macro-averaged across criteria, judges reached AC2 values of 0.69–0.86 and 0.62–0.87 on the nursing and report summarisation tasks, AC1 values of 0.52–0.88 on pass-or-fail error detection in MTB patient letters, and only 0.20–0.50 on pairwise comparison of discharge summaries.

For the two pointwise datasets, the variation across criteria was considerably greater than the variation across judges. For *comprehensible*, *organized*, *succinct*, and *stig.* *summary* all sat above 0.9 for every judge and spanned only 0.02–0.03 AC2. *Abstractive* scores ranged from −0.25 to 0.95, *clinical utility* scores ranged from −0.14 to 0.91, and *thorough* from 0.20 to 0.92 in nursing and 0.37 to 0.99 in reports. Median values for these criteria remained high (0.90, 0.78, 0.87, and 0.96, respectively) due to outliers. In the nursing dataset, the *accurate* and *cited* criteria were low for every judge (0.45–0.68 and 0.51–0.71, respectively), marking them as the only pointwise criteria for which no configuration surpassed substantial agreement. All judges achieved comparable macro-AC values on both datasets.

Pairwise comparison was the weakest paradigm for every judge, and the only paradigm in which no judge attained “substantial agreement” (AC1 > 0.6) at the macro level. Reliability was close to zero for *complete*, where judges ranged from −0.06 to 0.39 against a human reference of 0.51, and it was similarly weak for *clinical utility* (0.089–0.418 against 0.496). For the criterion humans agreed on most strongly (*human written*, 0.931), judges reliability values fluctuated between 0.115 and 0.814.

Reliability in the pass-or-fail paradigm was uniformly high, with a median AC1 of approximately 0.84, yet no judge significantly surpassed the human reference. Notably, Qwen3-235B-A22B-Instruct-2507-FP8/rule, which had the highest recall, had the lowest macro AC1 of 0.517.

### Robustness of the LLM-judges against consensus level, bias, prompt, and seeds

High alignment with one consensus reference is necessary but not sufficient for a trustworthy LLM judge. A judge whose verdicts shift with the candidate order, candidate identity, prompt wording, or random seed cannot be relied upon.

**Fig. 3:**
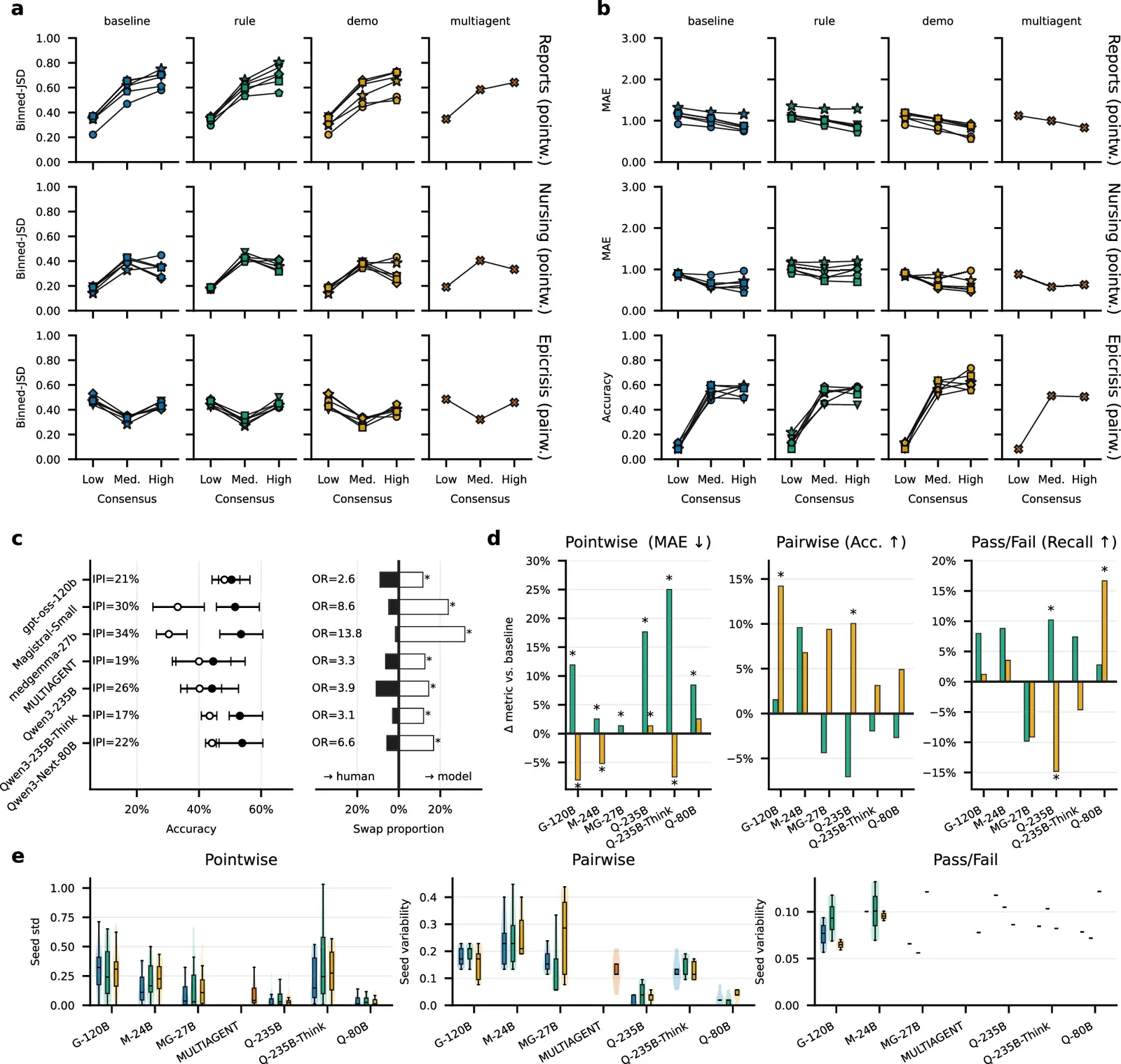
Robustness of the LLM-judges against consensus level, bias, prompt, and seeds. **a** Binned Jensen–Shannon divergence (Binned-JSD) between judge and human label distribution. One column per prompt configuration and one row per dataset (Reports pointwise, Nursing pointwise, Discharge summary pairwise). Within each cell, items are binned by how strongly the human raters agreed. “Low” covers items with ≤ 60% expert agreement on the majority label. “Med.” covers items with an agreement above 60% and below 100%. “High” covers items with 100% agreement. **b** Mean absolute error (MAE) against the human median for pointwise datasets. Accuracy against the human majority for the pairwise dataset. **c** Position and identity bias on the pairwise dataset. Intra-Pair Instability (IPI) quantifies the fraction of items with flipped votes after an order swap. Accuracy when the human-written summary is in the first or second position (left). Swap proportion toward the human/model candidate and odds ratio (OR) of model direction flips (right). An asterisk indicates Holm-corrected significance (*p* < 0.05) of the McNemar test. **d** Change in alignment metrics relative to each model’s baseline configuration under rule and demo prompts. **e** Per-judge seed influence across three random seeds. Asterisks in (**d**, **e**) mark significance at *P* < 0.05. Color denotes the prompt configuration (blue: baseline, green: rule, orange: demo, red: multiagent) and marker shape the judge model (circle: gpt-oss-120b, downward triangle: Qwen3-235B-A22B-Instruct-2507-FP8, star: Qwen3-235B-A22B-Thinking-2507-FP8, diamond: Qwen3-Next-80B-A3B-Instruct, pentagon: Magistral-Small-2509, square: medgemma-27b-it, cross: MULTIAGENT). Abbreviations: G-120B gpt-oss-120b, M-24B Magistral-Small-2509, MG-27B medgemma-27b-it, Q-235B Qwen3-235B-A22B-Instruct-2507-FP8, Q-235B-Think Qwen3-235B-A22B-Think-2507-FP8, Q-80B Qwen3-Next-80B-A3B-Instruct.

To investigate the effect of uncertainty between human raters, items were divided into three consensus groups based on the proportion of raters who agreed with the majority label (Fig. 3a, b). Within each bin, the binned Jensen–Shannon divergence (Binned-JSD) between the judge’s predicted distribution and the human label distribution was computed. Lower values correspond to greater similarity between judge and human distributions. Binned-JSD evaluates judges stricter where humans agree. High-consensus items concentrate human probability mass on a single label, so any deviation in the judge’s predicted rating yields a large divergence. In contrast, low-consensus items spread human ratings across several labels, and a single-rater judge falls inside that wider distribution more easily.

For pointwise judgements, divergence from the human reference distribution (Binned-JSD) increased from low- to high-consensus items under every prompt configuration (Fig. 3a). This shows that models and human raters disagree most where human raters agree most. This pattern is consistent with judges regressing towards the average rather than tracking the true score distribution. The pairwise paradigm follows another pattern. Judges rarely emit the tie label, so on low-consensus items where humans split between options, the judge distribution sits far from the human distribution. This produces high Binned-JSD alongside low accuracy within the same stratum. The section below also discusses the additional biases contributing to this gap. MAE (Fig. 3b) decreased modestly with increasing consensus, while pairwise accuracy increased sharply from low to medium consensus before plateauing.

For pairwise comparisons, each item was presented in both orders such that the human-written summary appeared once in the first slot and once in the second slot. Intra-pair instability (IPI), the fraction of items where the judge reversed its vote after the order swap, ranged from 17% for Qwen3-235B-A22B-Thinking-2507-FP8 to 34% for medgemma-27b-it. Accuracy against the consensus reference depended on which slot the human summary occupied. When the human summary appeared first, the accuracy ranged from 44% (Qwen3-235B-A22B-Instruct-2507-FP8, multiagent) to 54% (Qwen3-Next-80B-A3B-Instruct, medgemma-27b-it). When the human summary appeared second, the accuracy dropped for every judge, with the largest drops for medgemma-27b-it (54% → 30%) and Magistral-Small-2509 (52% → 33%), and the smallest drop for gpt-oss-120b (50% → 48%).

The combined vote share across both orders provides an estimate of the identity preference. McNemar tests on discordant pairs reached Holm-corrected significance for all six judges, with *p*-values of < 0.001 for all models, except for gpt-oss-120b and multiagent, for which *p* = 0.003. The asymmetry was unidirectional. Each judge flipped toward the model summary more often than toward the human summary, with swap-toward-model odds ratios of 13.8 (medgemma-27b-it) to 2.6 (gpt-oss-120b). The flip-toward-model proportion reached 32% for medgemma-27b-it and 24% for Magistral-Small-2509, compared with flip-toward-human proportions of only 2% and 5%, respectively.

Prompting shifts performance in opposite directions across evaluation paradigms (Fig. 3d). Prompt sensitivity was quantified as the raw change in the primary alignment metric relative to the baseline prompt. A positive value denotes increased MAE (worse pointwise performance) and increased accuracy or recall (better pairwise and pass/fail performance).

Rule augmentation significantly increased the MAE for all models, ranging from 1.5 pp for medgemma-27b-it to 25.0 pp for Qwen3-235B-A22B-Think-2507-FP8. The absolute increase was steeper for the larger models. Demonstration augmentation moved the MAE in the opposite direction for most models. It significantly reduced MAE for gpt-oss-120b ( − 8.0 pp), Magistral-Small-2509 ( − 5.5 pp), and Qwen3-235B-A22B-Think-2507-FP8 (−7.0 pp), with a small significant increase for Qwen3-235B-A22B-Instruct-2507-FP8 ( + 1.4 pp) and no significant change for Qwen3-Next-80B-A3B-Instruct.

The effect of demo prompts reversed on the pairwise and pass-or-fail paradigms. Demo prompting improved pairwise accuracy for all six models, significantly so for gpt-oss-120b ( + 14.2 points) and Qwen3-235B-A22B-Instruct-2507-FP8 ( + 10.1), whereas rule augmentation was inconsistent, improving Magistral-Small-2509 ( + 9.6) but reducing accuracy for medgemma-27b-it ( − 4.5), Qwen3-235B-A22B-Instruct-2507-FP8 ( − 7.0), and Qwen3-Next-80B-A3B-Instruct ( − 2.9). For pass-or-fail recall rule augmentation raised recall for gpt-oss-120b ( + 8.1), Magistral-Small-2509 ( + 8.9), Qwen3-235B-A22B-Instruct-2507-FP8 ( + 10.2) and Qwen3-235B-A22B-Think-2507-FP8 (+7.5) but lowered it for medgemma-27b-it ( − 9.8), while demo prompting produced both the largest significant gain (Qwen3-Next-80B-A3B-Instruct, +16.7) and significant loss (Qwen3-235B-A22B-Instruct-2507-FP8, −14.8). No prompting configuration improved all three paradigms for any model.

Each judge configuration was performed using three random seeds (Fig. 3e). In the pointwise task, Qwen3-Next-80B-A3B-Instruct (SD = 0.027) and Qwen3-235B-A22B-Instruct-2507-FP8 (SD = 0.034) were the most stable, followed by the multiagent ensemble (SD = 0.054). The reasoning models gpt-oss-120b, Magistral-Small-2509, and Qwen3-235B-A22B-Thinking-2507-FP8 produced markedly larger seed-to-seed variations on the Likert scale.

For the pairwise framework, stability was assessed using absolute vote variability across seeds. The pairwise was similar to the pointwise findings. Qwen3-Next-80B-A3B-Instruct and Qwen3-235B-A22B-Instruct-2507-FP8 reproduced their preference choices more precisely. The multiagent ensemble had a vote variability of 0.126, whereas the three remaining judges ranged between 0.17 (gpt-oss-120b) and 0.25 (Magistral-Small-2509).

In the pass-or-fail paradigm, however, the mean per-item seed disagreement rates ranged from 0.065 (gpt-oss-120b) to 0.103 (Qwen3-235B-A22B-Instruct-2507-FP8). Therefore, gpt-oss-120b was the most reproducible judge, and Qwen3-235B-A22B-Instruct-2507-FP8 was the least, reversing the pattern seen in the pointwise and pairwise paradigms, where the Qwen Instruct models were the most stable.

Prompting did not stabilize the judgments in a consistent direction. For some judges, the demo prompts increased seed dispersion relative to the baseline. In the pointwise task, gpt-oss-120b shifted from an SD of 0.286 to 0.363 under the demo prompt. In the pairwise task, medgemma-27b-it saw its vote variability increase from 0.166 to 0.259 under the demo configuration. For the other models, prompting modifications had little to no observable effects on output dispersion.

### Case Study: Substituting the expert panels with LLM-as-a-judge

**Fig. 4:**
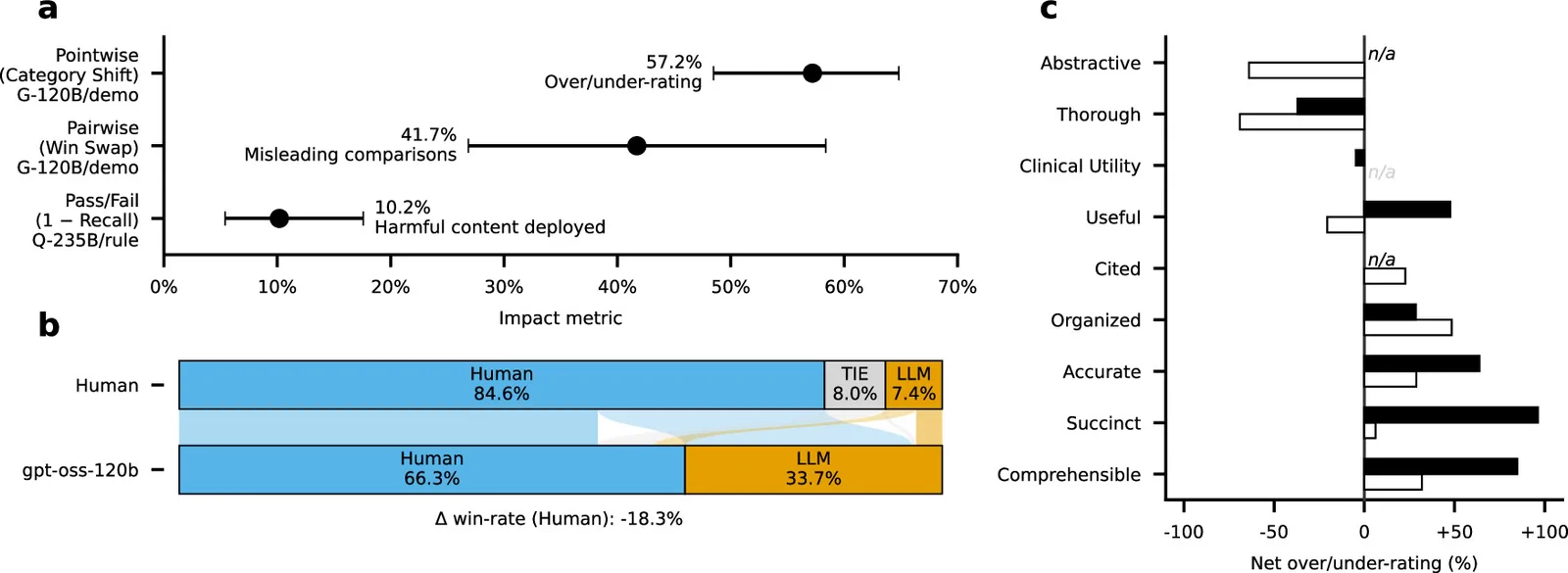
Case study of decision impact of using LLM-as-a-judge instead of the expert panels. **a** Aggregate impact metric for each paradigm under the best per-paradigm judge configuration. Point estimates with bootstrap 95% confidence intervals. Side labels translate each impact into its clinical consequence. Abbreviations: G-120B: gpt-oss-120b, Q-235B: Qwen3-235B-A22B-Instruct-2507-FP8. **b** Win-rate redistribution on the pairwise dataset. Human consensus (top) versus gpt-oss-120b/demo (bottom). **c** Net over-/under-rating of gpt-oss-120b/demo per criterion in the pointwise paradigm. The x-axis reports the net signed deviation as a percentage of items. Criteria absent from a given dataset are marked with “n/a”.

For every item rated by the human panel, the human consensus verdict was compared with the verdict the LLM judge would have returned, and the disagreement expressed in the assigned rating category (pointwise), the declared winner (pairwise), and the pass/fail safety gate (error detection) (Fig. 4). The human consensus decision serves as the counterfactual ground truth.

One judge selected based on the alignment with expert consensus was carried forward per paradigm. This selection is not a claim that these judges are the best available. Confidence intervals overlapped extensively across the model panel, and ranks were not statistically distinguishable. The analysis therefore describes what happens under a plausible, well-aligned choice of judge. gpt-oss-120b/demo was used in the pointwise and pairwise paradigm, and Qwen3-235B-A22B-Instruct-2507-FP8/rule in the pass-or-fail paradigm.

Substitution changed a large share of decisions in every paradigm. The judge assigned a different rating category from the human panel on 57.2% of item–criterion decisions (95% CI [48.5, 64.8]).

The direction of pointwise disagreement depended strongly on the criterion and dataset. Symmetric noise would produce similar rates of over- and under-rating within a criterion. Instead, for most criteria the net over/under-rating was high, meaning most disagreements fell on the same side (Fig. 4c). gpt-oss-120b/demo exhibits an almost unidirectional overrating profile. It inflated almost every criterion. The judge over-rated *succinct* on discharge reports (+96.2%; +1.31, 95% CI [1.19, 1.46]), *comprehensible* (+84.8% reports, +0.88, 95% CI [0.79, 0.96]; +32.0% nursing, +0.32, 95% CI [0.21, 0.42]), *accurate* (+63.8% reports, +0.66, 95% CI [0.53, 0.77]; +28.9% nursing, +0.56, 95% CI [0.32, 0.81), *organized* (+28.6% reports, +0.29, 95% CI [0.20, 0.37]; +48.5% nursing, +0.53, 95% CI [0.40, 0.65) and *cited* (+22.7% nursing, +0.49, 95% CI [0.28, 0.74]). It under-rated *thorough* (−69.1% nursing, −1.05, 95% CI [ − 1.23, −0.84]; −37.1% reports, −0.48, 95% CI [ − 0.63, −0.33]) and *abstractive* (−63.9% nursing, −0.99, 95% CI [ − 1.16, −0.80]). *Useful* reversed sign between datasets (+47.6% reports, +0.54, 95% CI [0.41, 0.69]; −20.6% nursing, −0.21, 95% CI [ − 0.37, −0.02]), so bias direction is not a fixed property of a criterion label. The largest single shift, *succinct* on discharge reports, moved the typical report by more than a full-scale point.

Pairwise substitution shifted the verdict against human-written text. The declared winner of a pairwise comparison changed on 41.7% of pairs (95% CI [26.9, 58.4]), and it varied by criterion, ranging from 22.9% for *human-written* to 54.3% for *clinical utility*. Among all text pairs, humans favored human-written discharge summaries 84.6% of the time, were tied 8.0% of the time, and favored LLM-written summaries 7.4%. However, gpt-oss-120b/demo favored human-written discharge summaries at 66.3% and LLM-written summaries at 33.7%, thus eliminating ties entirely.

The pass-or-fail paradigm has the most direct safety implications because it determines whether a section contains an error that potentially can cause patient harm. Recall of major and critical errors marked by the human expert was 0.90 for Qwen3-235B-A22B-Instruct-2507-FP8/rule, which resulted in a miss rate of 10% (95% CI [5%, 18%]).

## Discussion

This study shows that current open-weight LLM judges do not yet match expert review for German clinical text evaluation in the deployment-ready sense. The judges did not reproduce expert judgment of German clinical text on any of the four outcomes examined here. Alignment and reliability with expert consensus were dependent on criterion and context. Robustness analyses showed that judges diverged from the expert distribution most where experts were unanimous, showed substantial position bias, and preferred model-written to human-written text.

The decision-impact case study shows that substituting a judge for the expert panel changed 57.2% of pointwise rating categories (95% CI [48.5, 64.8]), reversed 41.7% of pairwise winners (95% CI [26.9, 58.4]), and left 10% of clinician-flagged major or critical errors undetected (95% CI [5, 18]), which means that human oversight remains necessary regardless of which open-weight judge is chosen. Reliability analyses further revealed that the configuration with the highest pass-or-fail recall (Qwen3-235B-A22B-Instruct-2507-FP8/rule) reached this recall at the cost of the low AC1, which reflects a high false-positive rate.

The rule-augmented prompt yielded the most counterintuitive finding of the audit. Prior work has proposed detailed operationalizations of each criterion as a remedy for evaluator drift^4,6^. However, this strategy decreased alignment and degraded reliability for most models. The likely mechanism concerns how judges acquire criterion meaning. Declarative rule text overrides the pre-trained semantics that the judge already attaches to terms such as *accurate*, *thorough*, or *succinct*, and defines a specification that the judge must apply on the fly. The demo prompt exposes the judge to canonical example judgments and, therefore, implicitly refines the existing pre-trained meaning rather than replacing it. This aligns with the well-established finding that LLMs excel at few-shot learning^23^. Although these gains did not close the gap to the expert panel for all criterions. While prompting strategies can potentially boost performance, their effects remain inconsistent and vary by model and paradigm, necessitating empirical validation before implementation.

Recent work has moved toward ensemble-based LLM judges (multiagent juries), which showed better agreement with human expert ratings than single-judge setups^24^ and have been adopted in domain-specific medical benchmarks, such as MedHELM^8^. Our results did not replicate the simple jury benefit. We attribute this provisionally to outliers in the multi-agent configuration, though further investigation is required. Under our current setup, the additional compute and orchestration costs of prompting multiple models for one rating are not justified by a measurable gain.

The signed-deviation analysis of the case study revealed an inversion of clinical priorities. The judge overrated *succinct*, *comprehensible*, and *accurate*, and underrated *thorough*. This pattern is consistent with the length and verbosity bias documented in general-domain judges^25,26^. This disparity may also stem from the highly specialized domain of clinical text, as LLM judges have been shown to align more closely with lay than expert criteria^21^. The clinical implications differ from those in the general domain because overrating *accurate* when rating clinical text has direct consequences for patient safety. Furthermore, increased verbosity hinders efficient communication, which is essential in highly busy clinical contexts.

Prior work in English clinical text evaluation^6,8^ has reported pointwise inter-rater reliability at or above the human baseline. While this audit mostly reproduces this pattern, it also shows that the same configurations diverge from the human panel when absolute alignment, consensus stratification, bias, and decision impact are analyzed.

Every judge in this audit favored the LLM-written candidate over the human-written reference. Qwen3-235B-A22B-Instruct-2507-FP8 produced the report, nursing, and discharge summaries and also served as a judge, so self-preference could plausibly explain part of its margin. However, other judges were more likely to swap their rating to the LLM-written than the authoring model, which rules out self-recognition as the dominant mechanism in this setting and separates our result from prior work^27^. We instead attribute the effect to a shared preference for surface features typical of LLM output, which the judges reward independent of provenance. medgemma-27b-it showed the largest position and identity bias by a large margin. Domain-adapted pretraining therefore offers no protection against the effect in this pool, though a single medical model does not let us separate the contribution of domain data from model scale and instruction tuning. Arena-style benchmarks that aggregate judge votes over mixed human and AI references will inflate apparent model quality, which should be considered when interpreting rankings from such benchmarks.

On items where humans themselves split, the human majority label is an unstable target (e.g., low AC1 for pairwise), and any judge that matches it perfectly would replicate that instability. Binned-JSD^12^ compares the full judge distribution to the full human distribution to show these distributional failures. The audit confirms that diagnostic value in a clinical setting, where the highest divergence concentrates on items at unanimous expert agreement, that is, the items on which downstream clinicians will most readily defer to the judge. Pooled correlations and pooled accuracies therefore offer a false floor of safety in clinical contexts. We argue that any future audit of clinical LLM judges should report stratified divergence by consensus level as the default, alongside the aggregate metrics that the current clinical literature has standardized.

The present audit suggests that within-paradigm validation may not reliably transfer. Judges that clear or approach human-human thresholds in one paradigm did not in others. Hospital governance bodies that select clinical AI based on a pointwise paradigm benchmark, therefore, lack the evidence base to extrapolate to deployment formats, such as safety guardrails.

The findings of this audit have direct implications for how LLM-as-a-judge can responsibly enter clinical infrastructure. First, as an offline benchmarking tool, an LLM judge with documented directional bias may still rank model versions consistently within a fixed evaluation regime, provided that the bias is reported alongside the score. Second, as a triage layer within a human-in-the-loop pipeline, the judge can flag candidate outputs for clinician review and reduce expert workload, where the pass-or-fail miss rate sets the lower bound on the rate of escaped errors that the human review must catch. Third, current judges fall short as standalone evaluators in deployed clinical systems. The pass-or-fail miss rate, directional overrating of the *accurate* criterion, and identity bias toward LLM output together rule out unsupervised use.

Across all three deployment patterns, the practical value of automated judging depends not only on alignment but also on whether evaluation outputs integrate into the surrounding clinical data environment. Hospital information systems already operate on structured, interoperable records for patient care, and judge output can plausibly be treated as a first-class artifact within the same infrastructure rather than as a separate logging stream. Such integration opens several opportunities, such as longitudinal model monitoring tied to individual patient encounters, governance dashboards that aggregate verdicts across clinical departments, and retrospective audits that connect an automated score to the exact prompt, model version, and clinical context in which it was produced.

One concrete entry point is the Fast Healthcare Interoperability Resources (FHIR) standard, whose Provenance resource can persist each automated evaluation along with the model identifier, prompt configuration, random seed, and timestamp. This audit trail aligns with the broader reproducibility and accountability expectations that hospital governance bodies and emerging regulations, such as the EU AI Act, place on high-risk clinical AI, and enables post-hoc analysis when downstream clinical decisions diverge from expectations.

This study has several limitations that highlight clear avenues for future research. First, the audit covers only open-weight models. Although closed-weight frontier models may produce different alignment patterns, the constraint of on-premises hospital deployment in the European Union limits the practical relevance of this comparison. Additionally, larger models were used in quantized formats to fit target hardware resources. This can affect calibration and confound model or model size effects. Second, our reliance on a clinician panel from a single institution suggests that findings may not fully account for inter-institutional variation. Future studies should employ multi-center designs to validate judge alignment across diverse clinical documentation conventions. Third, the high resource cost of expert annotation constrained our sample sizes. Future efforts should prioritize building larger, multi-annotator datasets to enhance the statistical resolution of subgroup analyses. Additionally, we did not evaluate more than one paradigm using the same texts, so future work should test judges across additional corpora to decouple paradigm-specific effects from dataset-specific variance. Fourth, the prompts tested here cover the dominant practical strategies but do not exhaust the space. For example, the multiagent configuration tested here covers only one ensemble strategy among many, with weighted voting, role-specific personas, and multi-turn debates left unexplored. A stronger claim about ensemble utility would require a dedicated ablation across aggregation methods and juror diversity. Furthermore, fine-tuning LLMs on larger human-annotated datasets could improve performance.

The decision-impact case study translates judges’ behavior into outcomes that hospital governance bodies can evaluate against specific deployment thresholds. We recommend that teams deploying an LLM judge verify it with the same rigor applied to training and calibrating human raters, including an explicit check to ensure that the judge measures the intended construct and reflects the quality priorities of the target setting. Consequently, future audits should adopt comparable substitution analyses rather than relying on aggregate alignment metrics alone. While the best-performing judges may currently serve as a triage layer to flag candidates for expert review, their use as standalone evaluators in benchmarks or safety guardrails requires further alignment improvements and rigorous validation. Because judge performance varies across contexts, datasets, models, prompts, and criteria, clinicians and governance bodies should carefully weigh the evaluation paradigm behind any benchmark before implementation, ensuring no single result is extrapolated without validation.

## Methods

**Fig. 5:**
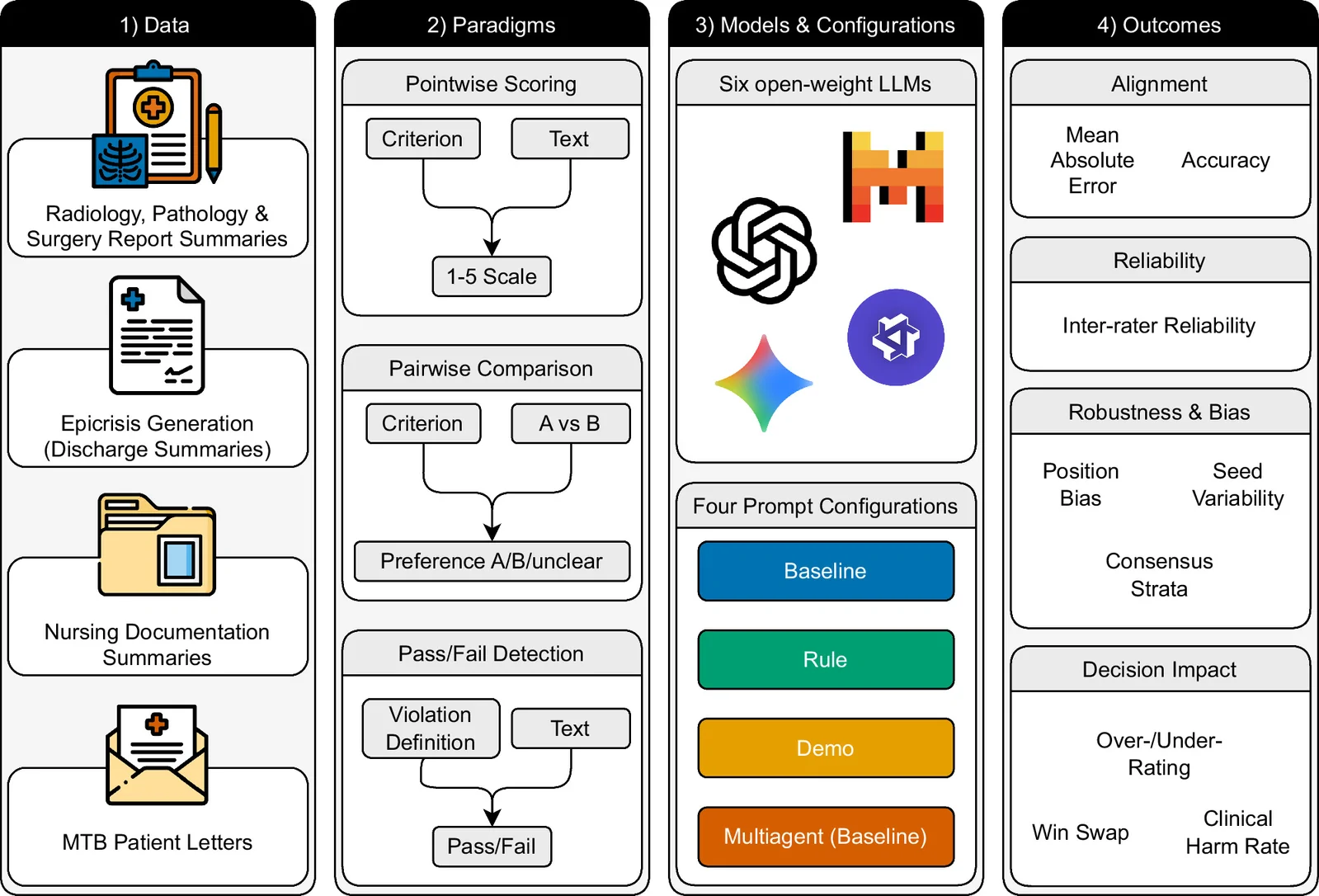
Study design for the cross-paradigm clinical audit of LLM-as-a-judge. 1) Data: Expert-annotated German medical text spanning radiology, pathology, and surgical report summaries, nursing documentation summaries, and molecular tumor board (MTB) patient letters. 2) Paradigms: Implements pointwise scoring (1–5 Likert scale), pairwise comparison (forced-choice ranking), and pass-or-fail detection (clinical safety guardrail checking). 3) Models and configurations: Six open-weight LLMs were evaluated across four prompt variants (baseline, rule, demo, and multiagent). 4) Outcomes: Measures judge performance through alignment with expert consensus, chance-corrected inter-rater reliability, algorithmic robustness against seed and position bias, and the impact on real-world clinical decisions. MAE mean absolute error, JSD Jensen–Shannon divergence, MTB molecular tumor board; Pass/Fail, pass-or-fail.

The study design is summarized in Fig. 5. Four expert-annotated German clinical datasets from previous studies served as the evaluation basis, each mapped to one of three evaluation paradigms. Six open-weight LLM judges were evaluated across four prompt configurations. Performance was assessed along four outcome dimensions: absolute alignment with expert consensus, chance-corrected inter-rater reliability, distributional and algorithmic robustness, and decision impact. This study followed the TRIPOD-LLM^28^ reporting standard (Suppl. Table 1).

### LLM-as-a-judge paradigms

This study covers three evaluation paradigms that together span the dominant output formats in current LLM-as-a-judge practice^4,9^. Each paradigm imposes a distinct demand on the judge, produces a different output format, and carries a different clinical risk profile in the event of failure (see Table 1).

**Table 1:** Demand, output format, and clinical risk profile of the three LLM-as-a-judge paradigms

Paradigm	Demand	Output	Risk if failed
Pointwise	Nuanced weighting of a criterion	Categorical score for a criterion	Over/underrating of clinical quality
Pairwise	Comparative reasoning	Preference ranking (A/B), with optional SAME/UNCLEAR	Misleading model comparisons
Pass-or-fail	Detection of explicit violations	Classification of error type and presence	Harmful content deployed or false alarms

In the pointwise paradigm, judges assign a categorical score to an item based on a single criterion. The judge receives a text, for example, an AI-generated radiology summary, together with a criterion such as “Is the summary succinct with economy of language”? The judge returns one label per criterion, often drawn from an ordinal scale. Pointwise scoring is the most common paradigm in clinical text evaluation and underlies rubric-based scoring frameworks such as PDSQI-9^6,10^.

In the pairwise paradigm, judges compare two texts based on comparative criteria. The judge receives both candidates, for example, two discharge summaries written for the same patient encounter, along with a criterion, such as, “which summary is better organized?”. The judge returns a preference or may also return a tie response (both equal). Pairwise comparison is widely used in head-to-head model evaluation and arena-style benchmarking^3,9^.

The pass-or-fail paradigm^9^ asks the judge to detect whether a defined violation is present in a single text. The judge receives a text, for example, a section of a patient letter, together with a violation definition, such as “Does the section contain an error?”. The judge returns a binary label indicating whether the violation occurred. This is also known as error detection^29^, annotation, or guardrails^30^.

### Datasets

This study employs four datasets from a large German academic university hospital. The four datasets cover four clinical documentation tasks widely used in everyday clinical work and are currently being targeted by research on clinical LLMs as candidates for automation. Each task carries a distinct documentation burden and maps onto one of the three evaluation paradigms in current LLM-as-a-judge practice.

For every dataset, the candidate texts and the expert ratings were fixed inputs to the audit. The expert panel rated each text once, and these ratings serve as the reference for all six judge models and all prompt configurations. A judge never sees another judge’s output, and the human reference does not change with the judge under test. Annotator and expert panel details can be found in Supplementary Text 1. The audit quantifies the extent to which the ratings agree on a set of predetermined texts, as opposed to evaluating the quality of the system that generated them.

All annotations were collected within separate primary studies^18–20^ that produced each dataset. The raters served as the expert evaluators in those studies. The report and discharge summary study^18^ is not yet published, and the rubrics are included in the Supplementary Text 2. Each rater worked independently. Annotations occurred in a structured web interface that displayed one item at a time and recorded raw responses without intermediate aggregation. Raters could not see other raters’ responses at any point.

The clinical report summaries dataset^18^ contains summaries of radiology, pathology, and surgery reports generated by Qwen3-235B-A22B-Instruct-2507-FP8. Thirty-five source documents per report type were entered into the analysis. Four clinicians rated radiology and pathology summaries, and three clinicians rated surgery summaries, against seven adapted PDSQI-9^10^ criteria. The dataset comprised 2695 ratings.

The nursing dataset^19^ contains forty-seven summaries of full nursing documentation and fifty summaries of nursing-relevant clinical endpoints, also generated by Qwen3-235B-A22B-Instruct-2507-FP8. Each received scores from at least four raters against ten adapted PDSQI-9 criteria, for a total of 3990 ratings. Two of the ten criteria flag the presence or absence of stigmatizing language and use a binary scale. The remaining eight criteria use the same five-point ordinal scale as the clinical report summaries dataset.

The discharge summary dataset^18^ contains thirty-five paired comparisons between human-written and Qwen3-235B-A22B-Instruct-2507-FP8-generated discharge summaries. Four clinicians evaluated each pair on five comparative criteria with response options left, both equal, and right, for a total of 700 ratings.

The molecular tumor board (MTB) patient letter dataset^20^ contains 710 segments drawn from these letters. The letters are lay explanations of MTB results and recommendations, generated by Llama-3.3-70B-Instruct^31^. Two oncologists independently annotated each section for clinical errors and assigned a severity grade to each error. The reference label per section is the union of major and critical errors flagged by either rater, so a section is considered to contain an error whenever at least one annotator identifies one. This rule reflects the asymmetric clinical risk that a missed error carries a higher consequence than a redundant flag.

The source material in all datasets came from routine clinical documentation. The inputs for clinical report summaries and MTB patient letters were single reports. The inputs for nursing documentation summaries and discharge summaries were hospital progress notes. Single reports tend to be more template-driven and focus on one topic, while progress notes are often repetitive, fragmented, and inconsistent in length and style. Every candidate text that a judge evaluated was produced under explicit content and formatting instructions defined in the respective primary study, and each instruction set specified a target audience. The clinical report summaries, discharge summaries, and nursing documentation summaries were generated for clinicians who specialize in the respective fields. Additional formatting instructions were used to generate the discharge summaries, matching the style of the human-written discharge summaries to maintain consistency between the compared texts. The MTB patient letters also followed clear formatting instructions; however, they addressed patients rather than clinical personnel, so their register was different. Of all the judged texts, the human-written discharge summaries demonstrate the greatest variation in wording and the elements of the clinical course that each author retained during compression.

Table 2 presents the dataset structure and rating volume. Full descriptive statistics, including text length, are reported in the Supplementary Tables 2–5.

**Table 2:** German clinical datasets per evaluation paradigm, with annotation source and total rating count (N) and inter-rater reliability

Paradigm	Dataset	Annotations	*N*	Inter-rater reliability
Pointwise	Clinical Report Summaries^18^ (Radiology, Pathology, Surgery)	Model-generated summaries rated with adapted PDSQI-9 criteria	2695	0.96 (Almost perfect agreement)
Pointwise	Nursing Documentation Summaries^19^	Model-generated summaries rated with adapted PDSQI-9 criteria	3990	0.80 (Substantial agreement) ordinal, 0.961 (Almost perfect agreement) binary
Pairwise	Discharge Summaries (epicrisis)^18^	Human and AI-generated summaries with expert judgments of comparative quality	700	0.78 (Substantial agreement)
Pass-or-fail	Molecular Tumor Board Patient Letters^20^	Union of clinician-annotated critical and major errors per segment	710	0.89 (Almost perfect agreement)

Measured by Gwet’s agreement coefficient interpreted using the Landis and Koch benchmark scale. AC of < 0.00: Worse than random (disagreement), 0.00–0.20: Slight agreement, 0.21–0.40: Fair agreement, 0.41–0.60: Moderate agreement, 0.61–0.80: Substantial agreement, 0.81–1.00: Almost perfect agreement.

### Judge models

Six open-weight large language models served as candidate judges. Table 3 lists the model identifiers, parameter counts, and quantization. The selection was based on the performance of the models on the Artificial Analysis Intelligence Index^32^ within the relevant size class, availability under a license compatible with deployment in the European Union, and computational feasibility on locally hosted hardware available in academic and hospital settings.

**Table 3:** Six open-weight judge models used in the audit, with parameter count, quantization, and reasoning capability

Creator	Model	Parameters	Quantization	Reasoning
Alibaba Cloud	Qwen3-235B-A22B-Instruct-2507-FP8^40^	235B	Fine-grained 8-bit floating-point (FP8)	no
Alibaba Cloud	Qwen3-235B-A22B-Think-2507-FP8^40^	235B	Fine-grained 8-bit floating-point (FP8)	yes
OpenAI	gpt-oss-120b^37^	120B	Micro-scaling Floating Point 4-bit (MXFP4)	yes
Alibaba Cloud	Qwen3-Next-80B-A3B-Instruct^40^	80B	n/a	no
Google	medgemma-27b-it^38^	27B	n/a	no
Mistral	Magistral-Small-2509^33,39^	24B	n/a	yes

Six models cover three deployment tiers. Qwen3-235B-A22B-Instruct-2507-FP8, Qwen3-235B-A22B-Thinking-2507-FP8, and gpt-oss-120b represent larger models often used in academic settings. Qwen3-235B-A22B-Instruct-2507-FP8 and Qwen3-235B-A22B-Thinking-2507-FP8 were quantized to fit target computing resources, while gpt-oss-120b comes natively in its MXFP4 quantization format. Qwen3-Next-80B-A3B-Instruct represents a mid-tier that balances parameter count against inference cost. Magistral-Small-2509 and medgemma-27b-it represent the smaller-model tier, whereas medgemma-27b-it has additional medical-domain pretraining. The six models also span three model families to reduce the risk that findings reflect the quirks of a single architecture. The deliberate exclusion of closed-weight frontier models reflects the constraint that on-premise hospital deployment in the European Union currently relies on open-weight models.

### Prompt configurations

The four configurations used in this audit are representative of the primary methods of LLM-as-a-judge prompting currently in use. The baseline prompt uses the rubric criteria as written in the annotation interface provided to human raters, without additional structuring or examples. It serves as a reference for unassisted judge performance. The rule-augmented (rule) prompt incorporates the criterion operationalizations from the codebook that was used to train human raters prior to annotation. Previous studies have demonstrated that this strategy can enhance criterion adherence^4,6^. The demonstration-based (demo) prompt adds example judgments that may align the automatic judge with human preferences^9,33^. The multiagent configuration aggregates baseline judgments from gpt-oss-120b, Magistral-Small-2509, and Qwen3-235B-A22B-Instruct-2507-FP8 via per-seed majority vote. This approach has been reported in the LLM-as-a-judge literature as effective at reducing variance and bias^4^. The same four configurations apply to all three paradigms. All prompt materials are based on the annotation tools and training documentation developed in the primary studies^18–20^ so that each judge condition drew on the same underlying source as the human raters. Full prompt texts for each configuration and task are provided in Supplementary Text 4.

### Inference settings and computation

The inference settings were set to the recommended parameters from each model card. Constrained decoding (llguidance^34^) was used to ensure consistent output formats. Detailed configurations are provided in the Zenodo repository. All inferences were run on locally hosted hardware via vLLM^35^ on NVIDIA A100 and H100 GPUs. Each judgment ran three times. The inputs were not modified between judges or configurations beyond the configuration-specific prompt. The total judge inference compute amounted to 61.1 GPU-hours.

### Evaluation framework and statistical analysis

All analyses operate at the level of item and criterion and aggregate by dataset, judge model, and prompt configuration. The four outcomes are absolute alignment, reliability, distributional robustness, and algorithmic robustness. The primary metrics and reference labels per outcome and paradigm are summarized in Table 4. Recall takes priority over precision in the pass-or-fail paradigm because the asymmetric clinical risk of missed errors outweighs the cost of over-flagging. Gwet’s coefficients are used in place of Cohen’s kappa because they are robust to the prevalence inflation observed on several criteria, in particular the binary stigmatizing-language items where positive labels are rare. Quadratic weights on AC2 penalize large category disagreements more than adjacent-category errors and match the ordinal structure of the PDSQI-9 scale. Binned-JSD compares the full distribution of judge scores against the full distribution of human scores per item without enforcing a single reference label and is reported per consensus stratum, defined as low at ≤ 60% agreement, medium at > 60% and < 100%, and high at 100%. Bootstrap 95% confidence intervals (*n* = 1,000 item-level resamples) accompany all primary point estimates. Full metric details and statistical test specifications are reported in Supplementary Text 3.

**Table 4:** Primary evaluation metrics by outcome, paradigm, and criterion type

Outcome	Paradigm and criterion type	Primary metric	Reference label
Alignment	Pointwise, ordinal	MAE	Human median
Alignment	Pointwise binary, Pairwise	Accuracy	Human majority
Alignment	Pass-or-fail	Recall	Union of clinician error flags
Reliability	Pointwise, ordinal	Gwet’s AC2, quadratic weights	Human panel
Reliability	Pointwise binary, Pairwise, Pass-or-fail	Gwet’s AC1	Human panel
Distributional robustness	Pointwise, Pairwise	Binned-JSD per consensus stratum	Human label distribution
Algorithmic robustness, position	Pairwise	IPI	Paired votes under reversed order
Algorithmic robustness, identity	Pairwise	Vote share to human vs LLM candidate	Aggregated votes across both orders
Algorithmic robustness, seed	All paradigms	Per-item seed disagreement rate	Three-seed replicates

Each outcome maps to a single primary metric per criterion type, with the reference label against which the metric is computed. Binned-JSD is stratified by expert consensus level (low ≤ 60%, medium > 60% and < 100%, high 100%). Algorithmic robustness components target the pairwise paradigm (position, identity) and all three paradigms (seed). Statistical inference procedures, bootstrap protocols, and full metric formulas are reported in Supplementary Text 3. *MAE* mean absolute error, *IPI* Intra-Pair Instability, *Binned-JSD* Binned Jensen-Shannon Divergence, *PDSQI-9* Provider Documentation Summarization Quality Instrument.

### Decision-impact case study

The four outcomes above quantify the properties of judge performance. However, they do not directly answer whether downstream clinical or scientific conclusions would change if a researcher replaced the human panel with an automated judge. The decision-impact case study addresses this question. For each item and criterion, the analysis compares human consensus decisions with the corresponding decisions from a selected judge.

The pointwise impact metrics are the category-flip rate, defined as the fraction of items for which the judge assigns a different ordinal category than the human majority, and the signed mean deviation, which captures the average direction of the flip (positive values indicate overrating, and negative values indicate underrating). The pairwise impact metrics are the winner-flip rate, defined as the fraction of pairs for which the judge selects a different winner than the human majority, and the change in win-rate for the AI-generated candidate under judge-based evaluation versus human-based evaluation. The pass-or-fail impact metric was the change in the section-level recall of clinical errors of major or critical severity.

### Ethics

The study was conducted in accordance with the Declaration of Helsinki. This study was approved by the Ethics Committee of the Medical Faculty of the University of Duisburg-Essen (approval number: 24-12063-BO). The ethics committee waived the requirement for informed consent, as the study involved only a retrospective analysis of pre-existing annotation data and no patient contact. The annotating clinicians participated under their existing employment contracts and received no additional compensation.

## Supplementary information

npj_digital_medicine_LLMaaJ_Supplement_revision.

## Data Availability

The datasets generated and/or analyzed during the current study are not publicly available due to patient privacy regulations and the data protection policies of the University Hospital Essen, as the underlying clinical data and annotations contain protected health information derived from the primary studies cited^18–20^. Access to the underlying data is available from the corresponding author on reasonable request, subject to approval by the responsible institutional ethics committee and execution of a data use agreement.

## Code availability

All custom code used to generate and analyze the datasets in this study is publicly available without restriction. The complete pipeline is archived at Zenodo (https://doi.org/10.5281/zenodo.23581372) and released under the MIT licence upon acceptance of this manuscript. Analyses were run with Python 3.10.12 using numpy 1.26.4, pandas 2.3.3, scipy 1.12.0, scikit-learn 1.7.2, statsmodels 0.14.6, irrCAC 0.4.4, joblib 1.5.3, matplotlib 3.10.8 and seaborn 0.13.2. Judge inference was served locally with vLLM v0.18.0.

## Declarations

### Use of generative AI

The authors declare the use of generative AI in the research and writing processes. According to the GAIDeT taxonomy^36^, the following tasks were delegated to generative AI tools under full human supervision: literature search and systematization, development of experimental or research protocols, code generation, code optimization, visualization, proofreading and editing, summarizing text, and reformatting. The generative AI tools used were ChatGPT, DeepL Write, Claude Code, and GitHub Copilot. The responsibility for the final manuscript lies entirely with the authors.
