# Supplementary material for: Cross-paradigm audit of large language model judges for clinical text evaluation
Source: NPJ Digit Med. 2026 Nov 4;9:850. doi: 10.1038/s41746-026-03443-2 (PMC13737126; doi:10.1038/s41746-026-03443-2)
Supplement: Supplementary file 1 [file 41746_2026_3443_MOESM1_ESM.pdf]

Supplementary Information

Cross-paradigm audit of large language model judges for clinical text evaluation

Tabea M. G. Pakull[1,2], Sina Warmer[3,4], Kamyar Arzideh[3,5], Thomas Hilser[6,7], Bernadette Hosters[8], Hendrik Damm[2,9], Felix Nensa[3,4], Martin Schuler[6,7,10], Katarzyna Borys[3,4], Peter A. Horn[1], René Hosch[3,4], and Christoph M. Friedrich[2,9,†]

1. Institute for Transfusion Medicine, University Hospital Essen, Essen, Germany
2. Department of Computer Science, University of Applied Sciences and Arts Dortmund (FHDO), Dortmund, Germany
3. Institute for Artificial Intelligence in Medicine (IKIM), University Hospital Essen, Essen, Germany
4. Institute of Diagnostic and Interventional Radiology and Neuroradiology, University Hospital Essen, Essen, Germany
5. Central IT Department, Data Integration Center, University Hospital Essen, Essen, Germany
6. West German Cancer Center (WTZ), University Hospital Essen, Essen, Germany
7. Department of Medical Oncology, University Hospital Essen, Essen, Germany
8. Department Nursing Development and Nursing Research, University Hospital Essen, Essen, Germany
9. Institute for Medical Informatics, Biometry and Epidemiology (IMIBE), University Hospital Essen, Essen, Germany
10. National Center for Tumor Diseases (NCT), NCT West, Essen, Germany

† **Corresponding Author:** Christoph M. Friedrich (ORCID: https://orcid.org/0000-0001-7906-0038, E-Mail: christoph.friedrich@fh-dortmund.de)

Keywords: LLM-as-a-judge, Text Evaluation, Clinical Documentation, Summarization, Lay Translation, AI Safety, Large Language Model

Supplementary Table 1: TRIPOD-LLM Reporting Checklist

(see following pages)

TRIPOD+LLM Checklist

Section / Topic	Item Number	Checklist Item	Research Design	LLM Task	Reported on Page
**Abstract**					
**Title**	2a	Identify the study as developing, fine-tuning, and/or evaluating the performance of an LLM, specifying the task, the target population, and the outcome to be predicted.	All	All	1
**Abstract**	2b	Provide a brief explanation of the healthcare context, use case and rationale for developing or evaluating the performance of an LLM.	E,H	All	2
**Objectives**	2c	Specify the study objectives, including whether the study describes LLMs development, tuning, and/or evaluation	All	All	2
**Methods**	2d	Describe the key elements of the study setting.	All	All	2
	2e	Detail all data used in the study, specify data splits and any selective use of data.	M,D,E	All	Not Required
	2f	Specify the name and version of LLM used.	All	All	N/A
	2g	Briefly summarize the LLM-building steps, including any fine-tuning, reward modeling, reinforcement learning with human feedback (RLHF), etc.	M,D	All	Not Required
	2h	Describe the specific tasks performed by the LLMs (e.g., medical QA, summarization, extraction), highlighting key inputs and outputs used in the final LLM.	All	All	2
	2i	Specify the evaluation datasets/populations used, including the endpoint evaluated, and detail whether this information was held out during training/tuning where relevant, and what measure(s) were used to evaluate LLM performance.	All	All	2
**Results**	2j	Give an overall report and interpretation of the main results.	All	All	2
**Discussion**	2k	Explicitly state any broader implications or concerns that have arisen in light of these results.	All	All	2
**Other**	2l	Give the registration number and name of the registry or repository (if relevant).	H	All	2
**Introduction**					

Section / Topic	Item Number	Checklist Item	Research Design	LLM Task	Reported on Page
**Background**	3a	Explain the healthcare context / use case (e.g., administrative, diagnostic, therapeutic, clinical workflow) and rationale for developing or evaluating the LLM, including references to existing approaches and models.	All	All	3-4
	3b	Describe the target population and the intended use of the LLM in the context of the care pathway, including its intended users in current gold standard practices (e.g., healthcare professionals, patients, public, or administrators).	E,H	All	3-4
**Objectives**	4	Specify the study objectives, including whether the study describes the initial development, fine-tuning, or validation of an LLM (or multiple stages).	All	All	4
**Methods**					
**Data**	5a	Describe the sources of data separately for the training, tuning, and/or evaluation datasets and the rationale for using these data (e.g., web corpora, clinical research/trial data, EHR data).	All	All	21-23
	5b	Describe the relevant data points and provide a quantitative and qualitative description of their distribution and other relevant descriptors of the dataset (e.g., source, languages, countries of origin)	All	All	21
	5c	Specifically state the date of the oldest and newest item of text used in the development process (training, fine-tuning, reward modeling) and in the evaluation datasets.	M,D,E,H	All	N/A
	5d	Describe any data pre-processing and quality checking, including whether this was similar across text corpora, institutions, and relevant sociodemographic groups.	All	All	N/A
	5e	Describe how missing and imbalanced data were handled and provide reasons for omitting any data.	M,D,E	All	Not Required

Section / Topic	Item Number	Checklist Item	Research Design	LLM Task	Reported on Page
**Analytical Methods**	6a	Report the LLM name, version, and last date of training or use during inference.	All	All	23-24
	6b	Specify the type of LLM architecture, and LLM building steps, including any hyperparameter tuning (e.g., temperature, length limits, penalties), prompt engineering, and any inference settings (e.g., seed, temperature, max token length) as relevant.	M,D,E	All	Not Required
	6c	Report details of LLM development process from text input to outcome generation, such as training, fine-tuning procedures, and alignment strategy (e.g., reinforcement learning, direct preference optimization, etc.) and alignment goals (e.g., helpfulness, honesty, harmlessness, etc.).	M,D	All	Not Required
	6d	Specify the initial and post-processed output of the LLM (e.g., probabilities, classification, unstructured text).	All	All	24-25
	6e	Provide details and rationale for any classification and how the probabilities were determined and thresholds identified.	All	C,OF	N/A
	6f	Include metrics that capture the quality of generative outputs, such as consistency, relevance, and accuracy, compared to gold standards.	All	QA,IR,DG,SS,MT	Not Required
	6g	Report the outcome metrics' relevance to downstream task at deployment time and correlation of metric to human evaluation of the text for the intended use.	E,H	All	25-26

Section / Topic	Item Number	Checklist Item	Research Design	LLM Task	Reported on Page
**LLM Output**	7a	Clearly define the outcome, how the LLM predictions were calculated (e.g., formula, code, object, API), and evaluation metrics.	E,H	All	24-26
	7b	If outcome assessment requires subjective interpretation, describe the qualifications of the assessors, any instructions provided, relevant information on demographics of the assessors, and inter-assessor agreement.	All	All	8-9, 21-23
	7c	Specify how performance was compared to other LLMs, humans, and other benchmarks or standards.	All	All	25-27
**Annotation**	8a	If annotation was done, report how text was labeled, including providing specific annotation guidelines with examples.	All	All	21-23, Supplementary
	8b	If annotation was done, report how many annotators labeled the dataset(s), including the proportion of data in each dataset that were annotated by more than 1 annotator.	All	All	21-23, Supplementary
	8c	If annotation was done, provide information on the background and experience of the annotators, and the inter-annotator agreement.	All	All	8-9, 21-23
**Prompting**	9a	If research involved prompting LLMs, provide details on the processes used during prompt design, curation, and selection.	All	All	24, Supplementary
	9b	If research involved prompting LLMs, report what data were used to develop the prompts.	All	All	24, Supplementary
**Summarization**	10	Describe any preprocessing of the data before summarization.	All	SS	Not Required
**Instruction Tuning / Alignment**	11	If instruction tuning/alignment strategies were used, what were the instructions and interface used for evaluation, and what were the characteristics of the populations doing evaluation?	M,D	All	Not Required
**Compute**	12	Report compute, or proxies thereof (e.g., time on what and how many machines, cost on what and how many machines, inference time, floating-point operations per second (FLOPs)), required to carry out methods.	M,D,E	All	Not Required

Section / Topic	Item Number	Checklist Item	Research Design	LLM Task	Reported on Page
**Ethics Approval**	13	Name the institutional research board or ethics committee that approved the study and describe the participant-informed consent or the ethics committee waiver of informed consent.	All	All	27
**Open Science**	14a	Give the source of funding and the role of the funders for the present study.	All	All	33
	14b	Declare any conflicts of interest and financial disclosures for all authors.	All	All	3-34
	14c	Indicate where the study protocol can be accessed or state that a protocol was not prepared.	H	All	N/A
	14d	Provide registration information for the study, including register name and registration number, or state that the study was not registered.	H	All	N/A
	14e	Provide details of the availability of the study data.	All	All	27-28
	14f	Provide details of the availability of the code to reproduce the study results.	All	All	28
**Public Involvement**	15	Provide details of any patient and public involvement during the design, conduct, reporting, interpretation, or dissemination of the study or state no involvement.	H	All	N/A
**Results**					

Section / Topic	Item Number	Checklist Item	Research Design	LLM Task	Reported on Page
**Participants**	16a	When using patient/EHR data, describe the flow of text/EHR/patient data through the study, including the number of documents/questions/participants with and without the outcome/label and follow-up time.	E,H	All	N/A
	16b	When using patient/EHR data, report the characteristics overall and, for each data source or setting, and for development/evaluation splits, including the key dates, key predictors, and sample size.	E,H	All	N/A
	16c	For LLM evaluation, show a comparison of the distribution of important predictors between development and evaluation data.	E,H	All	N/A
	16d	When using patient/EHR data, specify the number of participants and outcome events in each analysis (e.g., for LLM development, hyperparameter tuning, LLM evaluation).	E,H	All	N/A
**Performance**	17	Report LLM performance according to pre-specified metrics (see item 7a) and/or human evaluation (see item 7d).	All	All	5-15
**LLM Updating**	18	If applicable, report the results from any LLM updating, including the updated LLM and subsequent performance.	All	All	N/A
**Discussion**					
**Interpretation**	19a	Give an overall interpretation of the main results, including issues of fairness in the context of the objectives and previous studies.	All	All	15-19
**Limitations**	19b	Discuss any limitations of the study and their effects on any biases, statistical uncertainty, and generalizability.	All	All	18

Section / Topic	Item Number	Checklist Item	Research Design	LLM Task	Reported on Page
**Usability of the LLM in context**	19c	Describe any known challenges in using data for the specified task and domain context with reference to representation, missingness, harmonization, and bias.	E,H	All	16-18
	19d	Define the intended use for the implementation under evaluation, including the intended input, end-user, level of autonomy/human oversight.	E,H	All	17-19
	19e	If applicable, describe how poor quality or unavailable input data should be assessed and handled when implementing the LLM, i.e., what is the usability of the LLM in the context of current clinical care.	E,H	All	18-19
	19f	If applicable, specify whether users will be required to interact in the handling of the input data or use of the LLM, and what level of expertise is required of users.	E,H	All	18-19
	19g	Discuss any next steps for future research, with a specific view to applicability and generalizability of the LLM.	All	All	16-19

Supplementary Text 1: Annotator and Expert Panel Composition

Clinical Report Summaries[18]

Raters were medical professionals with specialty expertise matching the report type. Radiology and pathology reports were each rated by four raters; surgery reports by three. Raters used a custom annotation interface with the 7-criterion PDSQI-9-adapted rubric.

Nursing Handover Notes[19]

The rater panel consisted of ten nursing professionals with clinical experience ranging from 6 to 31 years (mean 13.7 years)[19]. All 97 notes were rated by at least four independent nursing staff members. Raters applied a 10-criterion adapted PDSQI-9 rubric.

Patient Letters[20]

Errors in patient letters were annotated by a panel of seven board-certified medical oncologists/hematologists from the WTZ. They served as annotators, with direct experience in complex MTB cases constituting relevant domain expertise. Each letter was independently rated by two clinicians. Annotations were made at section level, classifying each section as containing a major or critical error (fail) or not (pass). The two highest severity levels (ERHEBLICH/KRITISCH) from the error taxonomy triggered a fail label. The union of annotated errors from the raters were used.

Discharge Summary Pairs[18]

Four clinicians performed pairwise comparisons of AI-generated versus human-written discharge summaries. Each rater independently judged which text was better on each criterion, or declared a tie.

Supplementary Text 2: Evaluation Rubrics

Clinical Report Summaries Rubric

Criteria rated on a 1–5 integer scale. Scores are defined as follows[18]:

1 - Is the summary accurate in extraction (extractive summary)?

Very Inaccurate
Inaccurate
Fair
Accurate
Very Accurate

2 - Is the summary thorough without any omissions?

Very Insufficient
Insufficient
Fair
Thorough
Very Thorough

3 - Is the summary useful?

Not useful at all
Little Useful
Fairly Useful
Useful
Very Useful

4 - Is the summary organized?

Very Chaotic
Chaotic
Fairly Organized
Well Organized
Very Well Organized

5 - Is the summary comprehensible with clarity of language?

Very Unclear Language
Unclear Language
Fair Language
Clear Language
Very Clear Language

6 - Is the summary succinct with economy of language?

Very Long
Long
Medium Length
Short
Very Short

7 - How likely would you include the summary in a clinical letter?

Not at all
I would change many parts
I would change some parts
Likely
Very Likely

Nursing Handover Note Rubric

The nursing handover note evaluation, including rubric development, the PDSQI-9 adaptation, and the Delphi consensus rounds, was designed and conducted as part of a separate study[19]. We reused its rubric and ratings without modification. The full criterion definitions, scale anchors, and the two binary criteria are reported there. In brief, the rubric comprises eight criteria on an integer scale plus two binary criteria.

MTB Patient Letter Error Taxonomy

Each section of a patient letter is classified using the following error types and severity levels[20]. A section receives a FAIL label if it contains at least one MAJOR (Level 2) or CRITICAL (Level 3) error.

Error types:

- **FACTUAL ERROR**: A statement that is factually incorrect or contradicts the MTB protocol.
- **OMISSION**: A clinically relevant piece of information from the MTB protocol that is absent from the patient letter.
- **IRRELEVANCE**: Information present in the letter that is not clinically relevant or appropriate for a patient-facing letter.
- **LANGUAGE ERROR**: Inappropriate use of medical jargon, foreign terms, or unclear phrasing in a patient letter.

Severity levels:

- **MINOR (Level 1)**: Low clinical impact; no risk to patient safety or care decisions.
- **MAJOR (Level 2, ERHEBLICH)**: Significant clinical impact; could affect patient understanding or care decisions.
- **CRITICAL (Level 3, KRITISCH)**: High clinical impact; could cause direct patient harm or severe misunderstanding.

Discharge Summary Pairwise Criteria

Five criteria are judged pairwise (A better / B better / TIE)[18]:

1. human_written: Overall preference for the human-authored text.

2. completeness: Which text covers the clinical content more completely.

3. efficiency: Which text communicates the same clinical content more concisely.

4. organized: Which text is better organized for clinical readability.

5. clinical_utility: Which text has greater direct clinical utility

Supplementary Text 3: Statistical Methods and Metric Formulas

Alignment Metrics

Mean Absolute Error (MAE):

For pointwise paradigms, MAE against the human median is computed per item and averaged across items:

$$MAE = \frac{1}{N}\sum_{i=1}^{N}\left|\hat{y}_i - \tilde{y}_i^{human}\right|$$

where $\hat{y}_i$ is the LLM's score for item i and $\tilde{y}_i^{human}$ is the human median. The scale range is 1–5 for reports and nursing.

Pairwise accuracy (accuracy_clear):

For pairwise paradigms, accuracy is computed only on items with a clear human majority (>50%), excluding ambiguous 2-2 splits:

$$acc\_clear = \frac{\sum_i 1\left[\hat{v}_i = majority\left(v_i^{human}\right)\right]}{\sum_i 1[item\ i\ is\ not\ ambiguous]}$$

Clinical Harm Rate (CHR):

For the pass-or-fail paradigm, CHR is defined as 1 − recall (miss rate):

$$CHR = 1 - recall = 1 - \frac{TP}{FN+TP}$$

where FN = false negatives (failures missed by LLM, passed by LLM but failed by humans) and TP = true positives (failures correctly identified by LLM).

Reliability Metrics

Gwet's AC2 (quadratic weights) is used for ordinal (multi-category) agreement between LLM judge and individual human raters:

$$AC2 = \frac{P_o - P_e^{(q)}}{1 - P_e^{(q)}}$$

where P_o is observed agreement and $P_e^{(q)}$ is the chance-agreement correction using quadratic weights (appropriate for ordinal scales with clinically proportional distances).

Gwet's AC1 is used for binary and pairwise agreement:

$$AC1 = \frac{P_o - P_e^{(1)}}{1 - P_e^{(1)}}$$

where $P_e^{(1)}$ applies uniform category weights (appropriate for nominal/binary outcomes).

Consensus Binning

Items are stratified by the level of human expert consensus:

Bin	Definition	Interpretation
**High**	All raters agree (consensus = 100%)	Unambiguous items
**Medium**	Majority agreement > 60% and < 100%	Items with mild disagreement
**Low**	Majority agreement ≤ 60%	Genuinely ambiguous items

Threshold: majority_thresh = 0.75 determines a "clear majority" for pairwise items. Consensus bins: low = (0, 0.6], medium = (0.6, 1.0), high = [1.0, ∞).

Bootstrap Confidence Intervals

All reported confidence intervals are 95% bootstrap CIs based on 1000 item-level resamples (n_resamples = 1000). Items are resampled with replacement; each bootstrap replicate recomputes the metric on the resampled item set. The 2.5th and 97.5th percentiles define the CI bounds.

Macro-Averaging

All macro metrics average across criteria within a dataset, then across datasets, treating all criteria equally. Per-model summaries average across prompt configurations (baseline, rule, demo), unless otherwise specified.

Intra-Pair Instability (IPI)

For pairwise judgments, IPI measures position-order sensitivity. Each item is judged twice: once in the original order (A, B) and once in swapped order (B, A). IPI is the proportion of items where the LLM's preference reverses upon swapping:

$$IPI = \frac{1}{N}\sum_i 1\left[\hat{v}_i^{orig} \neq flip\left(\hat{v}_i^{swapped}\right)\right]$$

where $flip$ maps "A" to "B" and "B" to "A" (TIE maps to TIE).

Binned-JSD

Binned Jensen-Shannon Divergence (Binned-JSD) quantifies the divergence between the LLM's score

distribution and the human distribution, after discretizing scores into bins. A JSD of 0 indicates identical distributions; a JSD of 1 indicates completely non-overlapping distributions.

Binned-JSD is defined as follows[13]:

$$JS_b = \sum_{r \in R} \frac{|B_r|}{n} JS\left(P_r^h \parallel P_r^m\right) := \sum_{r \in R} \frac{|B_r|}{n} \left(\frac{1}{2} KL\left(P_r^h \parallel Q_r\right) + \frac{1}{2} KL\left(P_r^m \parallel Q_r\right)\right),$$

where $KL\left(P_r^h \parallel Q_r\right) = \sum_{c \in R} P_r^h(c) log_2\left(\frac{P_r^h(c)}{Q_r(c)}\right)$ is the Kullback-Leibler divergence and $Q_r(c) = \left(P_r^h(c) + P_r^m(c)\right)/2$ for $\forall c \in R$ is the average between human and machine PMFs, based on the definition of Jensen-Shannon distance (JS).

Supplementary Text 4: Prompt Texts

Model abbreviations used throughout: G-120B = gpt-oss-120b, M-24B = Magistral-Small-2509, MG-27B = medgemma-27b-it, Q-235B = Qwen3-235B-A22B-Instruct-2507, Q-80B = Qwen3-Next-80B-A3B-Instruct.

Three prompt configurations were used for all five models:

- Baseline: Criterion names and descriptions only; no rubric anchors.
- Rules: Full rubric with per-grade definitions for each criterion.
- Demo: Baseline prompt augmented with 2 randomly sampled few-shot examples from the same dataset (excluding the current item), each including the clinical text and a human expert's label as expected output.

A fourth configuration, Multiagent, aggregates baseline judgments from gpt-oss-120b, Magistral-Small-2509, and Qwen3-235B-A22B-Instruct-2507-FP8 by per-seed majority vote.

All prompts were constructed to mirror the criterion definitions and rubric anchors given to the expert evaluators in the respective primary studies[18,19,20], so that each judge received the same evaluation information as the human raters.

Clinical Report Summaries: Baseline Prompt

```
You are an expert clinical evaluator for summaries of clinical notes.

Read the following CLINICAL_NOTES. They were used to create a CLINICAL_SUMMARY.

<CLINICAL_NOTES>
{notes}
</CLINICAL_NOTES>

Read the following CLINICAL_SUMMARY, which is a summary of the above CLINICAL_NOTES for a
clinician with specialty {specialty}. Your task is to grade this CLINICAL_SUMMARY.

<CLINICAL_SUMMARY>
{summary_to_evaluate}
</CLINICAL_SUMMARY>

Grade the summary on the following criteria (each on a 1-5 scale):
- accurate: Is the summary accurate in extraction (extractive summary)?
- thorough: Is the summary thorough without any omissions?
- useful: Is the summary useful?
- organized: Is the summary organized?
- comprehensible: Is the summary comprehensible with clarity of language?
- succinct: Is the summary succinct with economy of language?
- clinical_utility: How likely would you include this summary in a clinical letter to the
patient?

Output your grades as a valid JSON object with all criteria as keys and integer grades (1-5)
as values.
```

Clinical Report Summaries: Rules Prompt (with Rubric)

You are an expert clinical evaluator for summaries of clinical reports.

Read the following CLINICAL_NOTES (report). They were used to create a CLINICAL_SUMMARY.

<CLINICAL_NOTES>
{notes}
</CLINICAL_NOTES>

Read the following CLINICAL_SUMMARY, which is a summary of the above CLINICAL_NOTES for a clinician with specialty {specialty}. Your task is to grade this CLINICAL_SUMMARY.

<CLINICAL_SUMMARY>
{summary_to_evaluate}
</CLINICAL_SUMMARY>

Use the following RUBRIC to grade the summary. Each dimension is evaluated independently.

<RUBRIC>

<accurate>
DESCRIPTION: Is the summary accurate in extraction (extractive summary)?
NOTE: Incorrect information can be fabrication (entirely made-up content not present in the original) or falsification (distorted facts). Each incorrect piece of information counts as a separate error.
EXCLUDE from this dimension: omissions of relevant information (covered by thorough).

GRADES:
1 = Very Inaccurate: Multiple major errors with overt falsifications or fabrications
2 = Inaccurate: A major error in assertion occurs with an overt falsification or fabrication
3 = Fair: At least one assertion contains a misalignment stated from a source note but wrong context
4 = Accurate: At least one assertion is misaligned to the provider source or timing but still factual
5 = Very Accurate: All assertions can be traced back to the notes
</accurate>

<thorough>
DESCRIPTION: The summary is complete and captures all clinically relevant information from the source.
NOTE: All essential information from the original report must be present. Both pertinent omissions (clearly important) and potentially pertinent omissions (may be relevant) are counted.
EXCLUDE from this dimension: correctness of extracted content (covered by accurate).

GRADES:
1 = Very Insufficient: More than one pertinent omission occurs
2 = Insufficient: One pertinent and multiple potentially pertinent omissions occur
3 = Fair: Only one pertinent omission occurs
4 = Thorough: Some potentially pertinent omissions occur
5 = Very Thorough: No pertinent or potentially pertinent omissions occur
</thorough>

<useful>
DESCRIPTION: All information in the summary is useful to the target provider.
NOTE: Assess whether all presented assertions are pertinent to the target clinician and appropriate in level of detail. Consider the clinician's specialty when judging relevance.

EXCLUDE from this dimension: how information is presented — order (organized), wording (comprehensible), conciseness (succinct).

GRADES:
1 = Not useful at all: No assertions are pertinent to the target user
2 = Little Useful: Some assertions are pertinent to the target user
3 = Fairly Useful: Assertions are pertinent but level of detail inappropriate (too detailed or not detailed enough)
4 = Useful: Not adding non-pertinent assertions but some assertions are potentially pertinent to target user
5 = Very Useful: Not adding non-pertinent assertions and level of detail is appropriate to targeted user
</useful>

<organized>
DESCRIPTION: The summary is well-formed and structured. Assertions are presented in logical order with coherent groupings.
NOTE: Assess both temporal order and systems/problem-based grouping. The structure should help the reader follow the clinical course.
EXCLUDE from this dimension: clarity of wording and language (comprehensible); conciseness (succinct).

GRADES:
1 = Very Chaotic: All assertions presented out of order and groupings incoherent (completely disorganized)
2 = Chaotic: Some assertions presented out of order OR grouping incoherent
3 = Fairly Organized: No change in order or grouping (temporal or systems/problem based) from original input
4 = Well Organized: Logical order or grouping (temporal or systems/problem based) for all assertions but not both
5 = Very Well Organized: All assertions made with logical order and grouping (temporal or systems/problem based) — completely organized
</organized>

<comprehensible>
DESCRIPTION: Clarity of language. The summary is clearly written and easy to understand by the target user.
NOTE: Assess language clarity, absence of ambiguity, correct use of technical terminology, and fluency. Language should be appropriate and familiar to the target clinician's specialty.
EXCLUDE from this dimension: redundancy and filler words (succinct).

GRADES:
1 = Very Unclear Language: Words and sentence structure are overly complex, inconsistent, with unfamiliar terminology
2 = Unclear Language: Any use of overly complex, inconsistent, or unfamiliar terminology to target user
3 = Fair Language: Unchanged choice of words from input with inclusion of overly complex terms when improvement possible
4 = Clear Language: Some inclusion of change in structure and terminology towards improvement
5 = Very Clear Language: Plain language completely familiar and well-structured to target user

ANSWER_OPTIONS:
1 = Very Unclear Language
2 = Unclear Language

```
    3 = Fair Language
    4 = Clear Language
    5 = Very Clear Language
</comprehensible>

<succinct>
    DESCRIPTION: Economy of language. The summary is concise without redundancy in syntax or
semantics.
    NOTE: Assess absence of unnecessary repetitions and filler words. The summary should be
to the point.
    EXCLUDE from this dimension: complex sentence structure or wrong word choice
(comprehensible); relevance of content (useful).

    GRADES:
    1 = Very Long: Too wordy across all assertions with redundancy in syntax and semantics
    2 = Long: More than one assertion has contextual semantic redundancy
    3 = Medium Length: At least one assertion has contextual semantic redundancy or multiple
syntactic assertions
    4 = Short: No syntax redundancy in assertions and at least one could have been shorter
in contextualized semantics
    5 = Very Short: All assertions are captured with fewest words possible and without any
redundancy in syntax or semantics
</succinct>

<clinical_utility>
    DESCRIPTION: How likely would you include this summary in a clinical letter to the
patient?
    NOTE: Assess the overall suitability of the summary for clinical handoff and patient
communication. This is a holistic judgment integrating accuracy, completeness, and
appropriateness for the target clinician and specialty.

    GRADES:
    1 = Not at all: Summary is unsuitable for a clinical letter
    2 = I would change many parts: Significant issues with accuracy, relevance, or clarity
    3 = I would change some parts: Some issues with relevance, completeness, or clarity that
require revision
    4 = Likely: Minor issues only, summary is largely suitable
    5 = Very Likely: Summary is accurate, complete, and fully appropriate for a clinical
letter
</clinical_utility>

</RUBRIC>

Output your grades as a valid JSON object with the following keys: "accurate", "thorough",
"useful", "organized", "comprehensible", "succinct", "clinical_utility".
All values should be integers between 1 and 5.
```

Nursing Handover Notes: Baseline Prompt

The criteria in this prompt are based on the rater evaluation codebook described by Hosters et al.[19].

```
You are an expert clinical evaluator for nursing documentation summaries.

Read the following CLINICAL_NOTES. They were used to create a CLINICAL_SUMMARY.
```

```
<CLINICAL_NOTES>
{notes}
</CLINICAL_NOTES>

Read the following CLINICAL_SUMMARY, which is a summary of the above CLINICAL_NOTES for
nursing staff. Your task is to grade this CLINICAL_SUMMARY.

<CLINICAL_SUMMARY>
{summary_to_evaluate}
</CLINICAL_SUMMARY>

Grade the summary on the following criteria (each on a 1-5 scale unless otherwise
specified):
- citation: Are citations present and appropriate? (1-5)
- accurate: Is the summary accurate in extraction? (1-5)
- thorough: Is the summary complete without omissions? (1-5)
- useful: Is all information useful to the target provider? (1-5)
- organized: Is the summary well-structured? (1-5)
- comprehensible: Is the language clear? (1-5)
- succinct: Is the language economical without redundancy? (1-5)
- abstraction: Is the summary abstracted and synthesized rather than a list of facts? (1-5)
- stig_original: Presence of stigmatizing language in the original note? (0=No, 1=Yes)
- stig_summary: Presence of stigmatizing language in the summary? (0=No, 1=Yes)

Output your grades as a valid JSON object with all criteria as keys and integer grades as
values.
```

Nursing Handover Notes: Rules Prompt (with Rubric)

The rubric anchors in this prompt are taken from the rater evaluation codebook described by Hosters et al.[19]. This prompt adds detailed rubric anchors for all eight ordinal criteria.

```
You are an expert clinical evaluator for nursing documentation summaries.



Read the following CLINICAL_NOTES. They were used to create a CLINICAL_SUMMARY.



<CLINICAL_NOTES>

{notes}

</CLINICAL_NOTES>



Read the following CLINICAL_SUMMARY, which is a summary of the above CLINICAL_NOTES for
nursing staff. Your task is to grade this CLINICAL_SUMMARY.



<CLINICAL_SUMMARY>
```

{summary_to_evaluate}

</CLINICAL_SUMMARY>

Use the following RUBRIC to grade the summary. Each dimension is evaluated independently.

For dimensions citation, accurate, and thorough: apply the WORSE of (a) the quantitative % correctness and (b) the clinical severity threshold. That is, even if % correctness would yield a higher grade, a single severe error that can gravely influence clinical decision-making caps the score at 2.

<RUBRIC>

<citation>

DESCRIPTION: Are citations present and appropriate? (Sind Zitate vorhanden und angemessen?)

NOTE: Evaluate (1) whether citations exist and are correctly reproduced — exact wording and timing must match the original note — and (2) whether each citation is correctly attributed to the right category or assertion. Count each incorrectly represented citation as one error, regardless of how many mistakes it contains.

EXCLUDE from this dimension: qualitative selection of which facts to cite.

GRADES (apply the worse of % correctness and clinical severity):

1 = 0–<25% of citations correct OR more than one severe error that can gravely influence clinical decision-making

2 = 25–<50% of citations correct OR at least one severe error that can gravely influence clinical decision-making

3 = 50–<75% of citations correct AND no severe error that can falsely influence clinical decisions

4 = 75–<100% of citations correct AND no severe error that can falsely influence clinical decisions

5 = 100% of citations correct AND no severe error that can falsely influence clinical decisions

</citation>

<accurate>

DESCRIPTION: Is the summary accurate in extraction (extractive summarization)? (Ist die Zusammenfassung in der Extraktion genau?)

NOTE: Incorrect information includes fabrication (entirely made-up content not present in the original) or falsification (distorted facts, e.g., "patient has Covid symptoms" rendered as "patient has Covid infection"). Each incorrect piece of information counts as a separate error.

EXCLUDE from this dimension: omissions of relevant information (covered by thorough); citation timing or attribution errors (covered by citation).

GRADES (apply the worse of % correctness and clinical severity):

1 = 0–<25% of assertions correct OR more than one severe error that can gravely influence clinical decision-making

2 = 25–<50% of assertions correct OR at least one severe error that can gravely influence clinical decision-making

3 = 50–<75% of assertions correct AND no severe error that can falsely influence clinical decisions

4 = 75–<100% of assertions correct AND no severe error that can falsely influence clinical decisions

5 = 100% of assertions correct AND no severe error that can falsely influence clinical decisions

</accurate>

<thorough>

DESCRIPTION: Is the summary thorough without any omissions? (Ist die Zusammenfassung vollständig ohne Auslassungen?)

NOTE: All essential information from the original source must be present. This includes both patient-specific information and relevant contextual factors (e.g., environmental fall or delirium risk factors, not only patient-related ones). Each missing relevant piece of information counts as one error.

EXCLUDE from this dimension: correctness of citation timing and attribution (covered by citation); correctness of extracted content (covered by accurate).

GRADES (apply the worse of % completeness and clinical severity):

1 = 0–<25% of essential information present OR more than one severe omission that can gravely influence clinical decision-making

2 = 25-<50% of essential information present OR at least one severe omission that can gravely influence clinical decision-making

3 = 50-<75% of essential information present AND no severe omission that can falsely influence clinical decisions

4 = 75-<100% of essential information present AND no severe omission that can falsely influence clinical decisions

5 = 100% of essential information present AND no severe omission that can falsely influence clinical decisions

</thorough>

<useful>

DESCRIPTION: Is the summary useful? (Ist die Zusammenfassung nützlich?)

NOTE: Assess whether the summary serves its intended purpose for the target audience — for nursing handover and surgical note use cases: all nursing-relevant information; for risk detection use cases (fall risk, delirium risk): presentation of relevant risk factors. Risk factors may be patient-related or environmental.

EXCLUDE from this dimension: how information is presented — order (organized), wording (comprehensible), conciseness (succinct), synthesis level (abstraction).

GRADES (degree of agreement with criterion):

1 = 0-<25% of the summary is useful to the target audience

2 = 25-<50% of the summary is useful to the target audience

3 = 50-<75% of the summary is useful to the target audience

4 = 75-99% of the summary is useful to the target audience

5 = 100% of the summary is useful to the target audience

</useful>

<organized>

DESCRIPTION: Is the summary organized? (Ist die Zusammenfassung gut strukturiert?)

NOTE: Assess logical order of contents for nursing staff so that the patient's situation is easy to understand. Sections should be sensibly delineated and not confusing.

EXCLUDE from this dimension: clarity of wording and language (comprehensible); conciseness (succinct); synthesis level (abstraction).

GRADES (degree of agreement with criterion):

1 = 0-<25% agreement with logical order and coherent structure

2 = 25-<50% agreement with logical order and coherent structure

3 = 50-<75% agreement with logical order and coherent structure

4 = 75-99% agreement with logical order and coherent structure

5 = 100% agreement — all assertions presented with logical order and coherent grouping

</organized>

<comprehensible>

DESCRIPTION: Is the summary comprehensible with clarity of language? (Ist die Zusammenfassung verständlich und klar formuliert?)

NOTE: Assess language clarity, absence of ambiguity, correct and appropriate use of technical terminology, and fluency. Language should be appropriate and familiar to the target user (nursing staff).

EXCLUDE from this dimension: stigmatizing language (stig_original, stig_summary); redundancy and filler words (succinct).

GRADES (degree of agreement with criterion):

1 = 0-<25% agreement with clear, unambiguous, appropriately worded language

2 = 25-<50% agreement with clear, unambiguous, appropriately worded language

3 = 50-<75% agreement with clear, unambiguous, appropriately worded language

4 = 75-99% agreement with clear, unambiguous, appropriately worded language

5 = 100% agreement — language completely clear, fluent, and familiar to target user

</comprehensible>

<succinct>

DESCRIPTION: Is the summary succinct with economy of language? (Ist die Zusammenfassung knapp und sprachlich ökonomisch?)

NOTE: Assess absence of unnecessary repetitions and filler words. The summary should be to the point.

EXCLUDE from this dimension: stigmatizing language (stig_original, stig_summary); overly complex or long sentences or wrong word choice (comprehensible); relevance of content (useful).

GRADES (degree of agreement with criterion):

1 = 0-<25% agreement — overly wordy with unnecessary repetitions and filler words throughout

2 = 25-<50% agreement with economy of language

3 = 50-<75% agreement with economy of language

4 = 75-99% agreement with economy of language

5 = 100% agreement — all assertions to the point, without any redundancy or filler words

</succinct>

<abstraction>

DESCRIPTION: Is the summary abstracted and synthesized rather than a list of facts? (Ist die Zusammenfassung abstrahiert und synthetisiert, statt nur einer Auflistung von Fakten?)

NOTE: Assess whether the summary interprets and synthesizes information rather than merely listing facts — e.g., through paraphrasing, drawing connections, or reasoning in the summary's own words.

EXCLUDE from this dimension: correctness of interpretations (accurate, useful); economy of language (succinct); stigmatizing language (stig_original, stig_summary).

GRADES (degree of agreement with criterion):

1 = 0-<25% agreement — summary is a mere enumeration of facts without synthesis or interpretation

2 = 25-<50% agreement with abstraction and synthesis

3 = 50-<75% agreement with abstraction and synthesis

4 = 75-99% agreement with abstraction and synthesis

5 = 100% agreement — fully synthesized and interpreted summary in the model's own words

</abstraction>

<stig_original>

DESCRIPTION: Is there presence of stigmatizing language in the original note? (Gibt es stigmatisierende Sprache in der Originalnotiz?)

NOTE: Language must be unbiased and respectful toward the patient. Any single instance of stigmatizing language results in a score of 1.

EXCLUDE from this dimension: correctness of clinical interpretation (accurate).

GRADES:

0 = No use of stigmatizing words

1 = Definite use of stigmatizing words

</stig_original>

<stig_summary>

DESCRIPTION: Is there presence of stigmatizing language in the summary? (Gibt es stigmatisierende Sprache in der Zusammenfassung?)

NOTE: Language must be unbiased and respectful toward the patient. Any single instance of stigmatizing language results in a score of 1.

EXCLUDE from this dimension: correctness of clinical interpretation (accurate).

GRADES:

0 = No use of stigmatizing words

1 = Definite use of stigmatizing words

</stig_summary>

</RUBRIC>

Output your grades as a valid JSON object with all criteria as keys and integer grades as values.

Use the following keys: "citation", "accurate", "thorough", "useful", "organized", "comprehensible", "succinct", "abstraction", "stig_original", "stig_summary".

MTB Patient Letter: Baseline Prompt

You are an expert clinical reviewer of AI-generated patient letters for the Molecular Tumor Board (MTB).

```
Read the following MTB_PROTOCOL (source) and the PATIENT_LETTER (AI-generated):

<MTB_PROTOCOL>
{source}
</MTB_PROTOCOL>

<PATIENT_LETTER>
{text}
</PATIENT_LETTER>

Review each numbered section of the patient letter for errors. A section receives FAIL if it
contains at least one major or critical error. Minor errors alone do not result in FAIL.

Output the result as a JSON object. Keys correspond to section numbers in the text (e.g.,
"section_0", "section_1", ...).

For each section, output an object with exactly these three fields in order:
1. "text": the exact section text as it appears in the PATIENT_LETTER
2. "comment": 1-2 sentences of reasoning explaining what error was found and why, or an
empty string if the section passes
3. "errors": "PASS" or "FAIL"
```

MTB Patient Letter: Rules Prompt (with Error Taxonomy)

```
You are an expert clinical reviewer of AI-generated patient letters for the Molecular Tumor
Board (MTB).

Read the following MTB_PROTOCOL (source) and the PATIENT_LETTER (AI-generated):

<MTB_PROTOCOL>
{source}
</MTB_PROTOCOL>

<PATIENT_LETTER>
{text}
</PATIENT_LETTER>

Use the following ERROR_TAXONOMY to evaluate each numbered section:

<ERROR_TAXONOMY>

ERROR TYPES:
- FACTUAL ERROR (FAKT): The text contains information that contradicts the MTB protocol
(internal) or established medical knowledge (external). Can mislead patients through
incorrect statements about diagnosis, therapy, or prognosis.
- OMISSION (AUSLASSUNG): Essential information is missing – either patient-specific details
from the MTB protocol (internal) or necessary background information for patient
understanding (external).
- IRRELEVANCE (IRRELEVANZ): The text contains superfluous information – irrelevant content
from the MTB protocol (internal) or general medical knowledge without relevance to the
specific case (external).
- LANGUAGE ERROR (SPRACHE): Typographical, grammatical, or stylistic errors without impact
on clinical content.
```

SEVERITY LEVELS:
- MINOR (GERING, Level 1): Limited impact on general understanding; terminological preferences without safety relevance.
- MAJOR (ERHEB, Level 2): Significant impact on patient understanding; errors in important medical facts or treatment plans.
- CRITICAL (KRIT, Level 3): Can cause patient harm through misunderstanding; significant inaccuracies in diagnosis, treatment, or prognosis; missing essential safety information.

EVALUATION RULE:
A section receives FAIL if it contains at least one error with severity MAJOR (ERHEB) or CRITICAL (KRIT).
Only MINOR (GERING) errors result in PASS.

</ERROR_TAXONOMY>

Output the result as a JSON object. Keys correspond to section numbers in the text (e.g., "section_0", "section_1", ...).

For each section, output an object with exactly these three fields in order:
1. "text": the exact section text as it appears in the PATIENT_LETTER
2. "comment": 1-2 sentences of reasoning explaining what error was found and why, or an empty string if the section passes
3. "errors": "PASS" or "FAIL"

Discharge Summary Pairs: Baseline Prompt

You are an expert clinical evaluator comparing two discharge summaries (Epikrisen).

Read the following SOURCE_TEXT on which both discharge summaries are based:

<SOURCE_TEXT>
{source_text}
</SOURCE_TEXT>

Compare the following two discharge summaries:

<DISCHARGE_SUMMARY_A>
{text_a}
</DISCHARGE_SUMMARY_A>

<DISCHARGE_SUMMARY_B>
{text_b}
</DISCHARGE_SUMMARY_B>

Grade the discharge summaries on the following criteria:
- human_written: Which discharge summary was written by a human?
- completeness: Which discharge summary is more complete in medical information content?
- efficiency: Which discharge summary presents the clinical course more efficiently?
- organized: Which discharge summary is better organized?
- clinical_utility: Which discharge summary would you rather include in a clinical letter?

For each criterion, respond with "A" if discharge summary A is better, "B" if discharge summary B is better, or "TIE" if they are equal.

Output your comparison as a valid JSON object with criteria as keys and "A", "B", or "TIE" as values.

Discharge Summary Pairs: Rules Prompt (with Rubric)

You are an expert clinical evaluator comparing two discharge summaries (Epikrisen).

Read the following SOURCE_TEXT on which both discharge summaries are based:

<SOURCE_TEXT>
{source_text}
</SOURCE_TEXT>

Compare the following two discharge summaries:

<DISCHARGE_SUMMARY_A>
{text_a}
</DISCHARGE_SUMMARY_A>

<DISCHARGE_SUMMARY_B>
{text_b}
</DISCHARGE_SUMMARY_B>

Use the following RUBRIC to evaluate the discharge summaries:

<RUBRIC>

<human_written>
QUESTION: Which discharge summary was written by a human?
DESCRIPTION: Assess which discharge summary you believe was written by a human rather than generated by AI.

GRADES:
A = Discharge summary A is more likely to have been written by a human
B = Discharge summary B is more likely to have been written by a human
TIE = Both discharge summaries are equally likely to have been written by a human
</human_written>

<completeness>
QUESTION: Which discharge summary has higher medical information content?
DESCRIPTION: Assess which discharge summary is more complete in terms of medical information. High medical information content means all information is included that influences patient care or is useful to know.

GRADES:
A = Discharge summary A has higher medical information content
B = Discharge summary B has higher medical information content
TIE = Both discharge summaries have equivalent medical information content
</completeness>

<efficiency>
QUESTION: Which discharge summary presents the clinical course more efficiently?
DESCRIPTION: Assess which discharge summary presents the treatment course more efficiently and concisely. This refers to the ratio of information content to text length.

GRADES:
A = Discharge summary A presents the clinical course more efficiently

B = Discharge summary B presents the clinical course more efficiently
TIE = Both discharge summaries present the clinical course equally efficiently
</efficiency>

<organized>
QUESTION: Which discharge summary is better organized?
DESCRIPTION: Assess which discharge summary is better organized. Well-organized means the summary is structured so that it helps the reader understand the patient's clinical course through logical order and coherent grouping.

GRADES:
A = Discharge summary A is better organized
B = Discharge summary B is better organized
TIE = Both discharge summaries are equally well organized
</organized>

<clinical_utility>
QUESTION: Which discharge summary would you rather include in a clinical letter?
DESCRIPTION: Assess which discharge summary you would rather include in a clinical letter. This refers to the suitability of the discharge summary for practical use in a clinical letter to the patient.

GRADES:
A = Discharge summary A is more suitable for a clinical letter
B = Discharge summary B is more suitable for a clinical letter
TIE = Both discharge summaries are equally suitable for a clinical letter
</clinical_utility>

</RUBRIC>

Output your comparison as a valid JSON object with criteria as keys and "A", "B", or "TIE" as values.

Dataset Descriptive Statistics

Supplementary Table 2 | Characteristics of the Clinical Report Summaries[18] dataset.

Statistic	Radiology	Pathology	Surgery
N documents (items)	35	35	35
N raters	4	4	3
N rating events	980	980	735
Evaluation paradigm	Pointwise	Pointwise	Pointwise
Scale	1–5 (7 criteria)	1–5 (7 criteria)	1–5 (7 criteria)
Human-human AC2 (macro)	0.853 [0.836, 0.870]	0.868 [0.849, 0.886]	0.943 [0.929, 0.957]
Source note length: words, mean (range)	108 (33–326)	144 (48–501)	439 (167–838)
Source note length: chars, mean (range)	935 (283–2888)	1198 (407–4330)	3589 (1418–6627)
Summary length: words, mean (range)	20 (9–42)	22 (10–39)	37 (22–52)
Summary length: chars, mean (range)	196 (87–373)	217 (97–433)	377 (212–546)

Criteria: clinical utility, accuracy, thoroughness, usefulness, succinctness, comprehensibility, organization.

Supplementary Table 3 | Characteristics of the Nursing[19] dataset.

Statistic	Summary subtask	Endpoints subtask	Combined
N items	47	50	97
N raters	≥4	≥4	-
N rating events	1890	2100	3990
Evaluation paradigm	Pointwise	Pointwise	Pointwise
Criteria	-	-	10
Human-human AC2 (macro)	0.712 [0.692, 0.732]	0.755 [0.736, 0.774]	0.733
Source note length: words, mean (range)	-	-	322 (7–888)
Source note length: chars, mean (range)	-	-	2582 (60–6897)
Summary length: words, mean (range)	-	-	254 (87–569)
Summary length: chars, mean (range)	-	-	2063 (841–4403)

Criteria: accuracy, thoroughness, citation, abstraction, usefulness, succinctness, comprehensibility, organization (1–5 scale), plus stig_original and stig_summary (binary 0/1).

Supplementary Table 4 | Characteristics of the MTB Patient Letters[20] dataset.

Statistic	Value
N letters	50
N document sections	710 (across all letters)
Evaluation paradigm	Pass-or-fail
Annotated aspects	Major/critical errors per section (union)
Source letter length: words, mean (range)	1004 (582–1493)
Source letter length: chars, mean (range)	8279 (4942–11742)
Patient letter length: words, mean (range)	537 (417–662)
Patient letter length: chars, mean (range)	4068 (3168–5014)

Each letter is divided into clinical sections (e.g., diagnosis, therapy plan, follow-up); each section receives an independent pass/fail label.

Supplementary Table 5 | Characteristics of the Discharge Summary[18] Pairwise dataset.

Statistic	Value
N document pairs	35
N raters per pair	4
N rating events	700
Evaluation paradigm	Pairwise comparison
Criteria	5
Ambiguous pairs (2-2 splits)	15.6 per criterion on average
Human-human AC1 (pairwise macro)	0.567 [0.526, 0.608]
Source text length: words, mean (range)	143 (6–898)
Source text length: chars, mean (range)	1071 (30–6721)
Candidate A length: words, mean (range)	176 (46–564)
Candidate A length: chars, mean (range)	1502 (389–4633)
Candidate B length: words, mean (range)	179 (46–431)
Candidate B length: chars, mean (range)	1523 (383–3591)

Complete Alignment Results

The following tables report alignment metrics for all model–prompt combinations across all four datasets. Values are reported as point estimate [95% CI].

Model abbreviations: G = G-120B (gpt-oss-120b), M = M-24B (Magistral-Small-2509), MG = MG-27B (medgemma-27b-it), Q235 = Q-235B (Qwen3-235B-A22B), Q80 = Q-80B (Qwen3-Next-80B), MA = MULTIAGENT.

Supplementary Table 6 | Clinical Report Summaries: MAE by Criterion (lower = better) Scale: 1–5 (range 1–4). Human median is the reference. Note: succinct is systematically high across all judges (LLMs consistently assign higher scores than humans on succinctness).

	clinical_utility	accurate	thorough	useful	succinct	comprehensible	organized
G-120B - baseline	0.78 [0.66, 0.91]	0.77 [0.68, 0.86]	0.94 [0.81, 1.07]	0.74 [0.61, 0.86]	1.39 [1.27, 1.52]	0.90 [0.82, 0.98]	0.35 [0.26, 0.44]
G-120B - rule	1.03 [0.90, 1.17]	1.01 [0.95, 1.08]	0.93 [0.79, 1.08]	0.80 [0.68, 0.94]	1.40 [1.28, 1.52]	1.00 [0.94, 1.07]	0.85 [0.76, 0.94]
G-120B - demo	0.68 [0.55, 0.81]	0.75 [0.65, 0.84]	0.62 [0.50, 0.75]	0.70 [0.59, 0.81]	1.35 [1.23, 1.49]	0.87 [0.79, 0.95]	0.36 [0.27, 0.45]
M-24B - baseline	0.84 [0.72, 0.98]	1.00 [0.93, 1.09]	0.27 [0.20, 0.37]	1.20 [1.10, 1.33]	1.40 [1.28, 1.52]	1.03 [0.95, 1.10]	0.99 [0.93, 1.05]
M-24B - rule	0.67 [0.55, 0.80]	1.04 [0.97, 1.11]	0.33 [0.25, 0.42]	1.20 [1.09, 1.31]	1.05 [0.92, 1.19]	1.05 [0.98, 1.12]	0.90 [0.83, 0.99]
M-24B - demo	0.93 [0.80, 1.06]	0.74 [0.64, 0.84]	0.41 [0.31, 0.50]	1.01 [0.89, 1.15]	1.22 [1.10, 1.35]	0.78 [0.68, 0.88]	0.77 [0.68, 0.87]
MG-27B - baseline	1.31 [1.19, 1.41]	1.06 [1.00, 1.13]	0.42 [0.31, 0.52]	1.25 [1.15, 1.38]	1.40 [1.28, 1.52]	1.05 [0.98, 1.12]	0.89 [0.81, 0.95]
MG-27B - rule	1.09 [0.96, 1.20]	1.06 [1.00, 1.13]	0.27 [0.20, 0.35]	1.28 [1.19, 1.40]	1.40 [1.28, 1.52]	1.05 [0.98, 1.12]	0.73 [0.64, 0.82]
MG-27B - demo	1.33 [1.22, 1.43]	1.06 [1.00, 1.13]	0.38 [0.29, 0.49]	1.26 [1.17, 1.39]	1.40 [1.28, 1.52]	1.05 [0.98, 1.12]	0.88 [0.79, 0.96]
MULTIAGENT - multiagent	1.01 [0.89, 1.13]	1.05 [0.99, 1.12]	0.41 [0.32, 0.51]	1.08 [0.95, 1.20]	1.40 [1.28, 1.52]	1.05 [0.98, 1.12]	0.98 [0.92, 1.05]
Q-235B - baseline	1.02 [0.90, 1.14]	1.06 [1.00, 1.13]	0.40 [0.31, 0.51]	1.09 [0.97, 1.21]	1.40 [1.28, 1.52]	1.05 [0.98, 1.12]	0.98 [0.92, 1.05]
Q-235B - rule	0.76 [0.64, 0.86]	1.03 [0.96, 1.10]	0.86 [0.75, 0.96]	1.15 [1.02, 1.27]	1.37 [1.25, 1.50]	1.04 [0.97, 1.11]	0.97 [0.90, 1.03]
Q-235B - demo	1.21 [1.10, 1.33]	1.06 [1.00, 1.13]	0.27 [0.19, 0.36]	1.22 [1.11, 1.35]	1.39 [1.27, 1.51]	1.04 [0.97, 1.11]	0.98 [0.92, 1.05]
Q-235B-Think - baseline	2.65 [2.55, 2.76]	0.90 [0.82, 0.98]	0.59 [0.50, 0.68]	1.08 [0.97, 1.20]	1.39 [1.27, 1.52]	1.01 [0.94, 1.08]	0.97 [0.91, 1.03]
Q-235B-Think - rule	1.85 [1.70, 2.01]	1.04 [0.96, 1.13]	1.67 [1.47, 1.86]	1.22 [1.13, 1.34]	1.34 [1.22, 1.47]	1.02 [0.95, 1.08]	0.97 [0.91, 1.03]
Q-235B-Think - demo	0.92 [0.77, 1.07]	0.85 [0.75, 0.93]	0.59 [0.49, 0.69]	1.06 [0.96, 1.17]	1.38 [1.26, 1.51]	1.00 [0.93, 1.06]	0.95 [0.88, 1.02]
Q-80B - baseline	1.28 [1.16, 1.39]	1.03 [0.96, 1.11]	0.38 [0.30, 0.48]	1.22 [1.12, 1.34]	1.40 [1.28, 1.52]	1.05 [0.98, 1.12]	0.99 [0.93, 1.05]
Q-80B - rule	0.94 [0.81, 1.06]	1.03 [0.96, 1.10]	0.56 [0.47, 0.65]	1.28 [1.19, 1.40]	1.39 [1.28, 1.51]	1.05 [0.98, 1.12]	0.93 [0.86, 1.00]
Q-80B - demo	1.30 [1.19, 1.40]	1.06 [1.00, 1.13]	0.38 [0.30, 0.48]	1.24 [1.14, 1.36]	1.40 [1.28, 1.52]	1.05 [0.98, 1.12]	0.99 [0.93, 1.05]

Supplementary Table 7 | Nursing Handover Notes: MAE by Criterion (lower = better) Scale: 1–5. Human median is the reference. Note: stig_original and stig_summary (binary) use accuracy instead of MAE.

	accurate	thorough	citation	abstraction	useful	succinct	comprehensible	organized	stig_original	stig_summary
G-120B - baseline	1.11 [0.97, 1.26]	1.31 [1.14, 1.47]	1.17 [0.96, 1.39]	1.29 [1.13, 1.43]	0.78 [0.66, 0.90]	0.40 [0.31, 0.49]	0.34 [0.25, 0.42]	0.54 [0.44, 0.66]	1.00 [1.00, 1.00]	0.99 [0.95, 1.00]
G-120B - demo	1.04 [0.88, 1.22]	1.32 [1.17, 1.46]	0.95 [0.78, 1.17]	1.07 [0.92, 1.23]	0.75 [0.64, 0.86]	0.43 [0.35, 0.53]	0.31 [0.23, 0.39]	0.52 [0.42, 0.64]	1.00 [1.00, 1.00]	0.99 [0.95, 1.00]
G-120B - rule	1.35 [1.18, 1.55]	1.96 [1.79, 2.12]	1.07 [0.89, 1.26]	1.37 [1.21, 1.53]	1.16 [1.04, 1.30]	0.31 [0.24, 0.39]	0.26 [0.20, 0.35]	0.52 [0.43, 0.63]	1.00 [1.00, 1.00]	0.99 [0.95, 1.00]
M-24B - baseline	1.42 [1.24, 1.64]	0.59 [0.50, 0.70]	1.35 [1.12, 1.60]	0.58 [0.48, 0.67]	0.80 [0.70, 0.93]	0.43 [0.35, 0.52]	0.26 [0.19, 0.34]	0.57 [0.47, 0.69]	0.95 [0.89, 0.98]	0.99 [0.95, 1.00]
M-24B - demo	1.37 [1.18, 1.59]	0.71 [0.59, 0.84]	1.27 [1.06, 1.51]	0.51 [0.42, 0.61]	0.82 [0.72, 0.94]	0.52 [0.42, 0.62]	0.28 [0.21, 0.36]	0.58 [0.48, 0.70]	1.00 [1.00, 1.00]	0.99 [0.95, 1.00]
M-24B - rule	1.43 [1.24, 1.63]	0.86 [0.75, 0.96]	1.31 [1.09, 1.55]	1.09 [0.97, 1.22]	0.67 [0.57, 0.80]	0.65 [0.57, 0.72]	0.40 [0.32, 0.49]	0.55 [0.45, 0.65]	0.98 [0.93, 1.00]	0.99 [0.95, 1.00]
MG-27B - baseline	1.42 [1.25, 1.64]	0.70 [0.60, 0.84]	1.26 [1.07, 1.49]	0.69 [0.58, 0.81]	0.81 [0.71, 0.95]	0.59 [0.49, 0.69]	0.26 [0.19, 0.34]	0.57 [0.47, 0.69]	0.98 [0.94, 1.00]	0.97 [0.92, 0.99]
MG-27B - demo	1.46 [1.27, 1.66]	0.65 [0.56, 0.79]	1.23 [1.03, 1.44]	0.57 [0.46, 0.70]	0.87 [0.75, 0.99]	0.43 [0.35, 0.55]	0.26 [0.19, 0.34]	0.57 [0.47, 0.69]	1.00 [1.00, 1.00]	0.99 [0.95, 1.00]
MG-27B - rule	1.46 [1.27, 1.66]	0.72 [0.61, 0.82]	1.12 [0.94, 1.31]	1.58 [1.35, 1.78]	0.85 [0.74, 0.97]	0.40 [0.31, 0.52]	0.26 [0.19, 0.34]	0.57 [0.47, 0.69]	1.00 [1.00, 1.00]	0.99 [0.95, 1.00]
MULTIAGENT - multiagent	1.38 [1.21, 1.60]	0.64 [0.54, 0.75]	1.37 [1.14, 1.61]	0.54 [0.43, 0.64]	0.85 [0.74, 0.97]	0.37 [0.28, 0.46]	0.26 [0.19, 0.34]	0.57 [0.47, 0.69]	0.96 [0.90, 0.99]	0.99 [0.95, 1.00]
Q-235B - baseline	1.39 [1.22, 1.62]	0.64 [0.54, 0.75]	1.37 [1.14, 1.61]	0.45 [0.36, 0.55]	0.85 [0.74, 0.97]	0.37 [0.29, 0.48]	0.26 [0.19, 0.34]	0.57 [0.47, 0.69]	0.76 [0.67, 0.84]	0.99 [0.95, 1.00]
Q-235B - demo	1.39 [1.21, 1.61]	0.69 [0.59, 0.81]	1.24 [1.04, 1.46]	0.53 [0.44, 0.62]	0.76 [0.65, 0.89]	0.37 [0.28, 0.48]	0.26 [0.19, 0.34]	0.57 [0.47, 0.69]	0.92 [0.85, 0.96]	0.99 [0.95, 1.00]
Q-235B - rule	1.43 [1.23, 1.62]	1.73 [1.53, 1.92]	1.09 [0.88, 1.30]	2.39 [2.18, 2.60]	0.70 [0.60, 0.81]	0.35 [0.27, 0.45]	0.26 [0.19, 0.34]	0.57 [0.47, 0.69]	0.99 [0.96, 1.00]	0.99 [0.95, 1.00]
Q-235B-Think - baseline	1.12 [0.95, 1.29]	1.14 [0.99, 1.27]	1.01 [0.82, 1.21]	0.60 [0.49, 0.71]	0.77 [0.64, 0.89]	0.38 [0.30, 0.48]	0.27 [0.20, 0.35]	0.48 [0.39, 0.59]	0.98 [0.94, 1.00]	0.99 [0.95, 1.00]
Q-235B-Think - demo	1.04 [0.89, 1.20]	1.33 [1.18, 1.51]	1.01 [0.86, 1.19]	1.03 [0.88, 1.17]	0.98 [0.86, 1.12]	0.37 [0.29, 0.46]	0.28 [0.21, 0.37]	0.45 [0.36, 0.54]	1.00 [1.00, 1.00]	0.99 [0.95, 1.00]
Q-235B-Think - rule	1.29 [1.11, 1.45]	2.19 [2.03, 2.34]	1.25 [1.07, 1.47]	2.20 [1.97, 2.39]	0.84 [0.71, 0.97]	0.38 [0.30, 0.49]	0.27 [0.21, 0.35]	0.58 [0.49, 0.70]	1.00 [1.00, 1.00]	0.99 [0.95, 1.00]
Q-80B - baseline	1.40 [1.22, 1.62]	0.67 [0.57, 0.80]	1.39 [1.15, 1.64]	0.52 [0.42, 0.62]	0.86 [0.75, 0.98]	0.40 [0.32, 0.49]	0.28 [0.22, 0.36]	0.57 [0.47, 0.69]	0.96 [0.91, 0.99]	0.99 [0.95, 1.00]
Q-80B - demo	1.41 [1.22, 1.62]	0.72 [0.60, 0.86]	1.37 [1.13, 1.61]	0.40 [0.33, 0.51]	0.86 [0.75, 0.99]	0.40 [0.32, 0.51]	0.28 [0.22, 0.36]	0.57 [0.47, 0.69]	0.94 [0.88, 0.98]	0.99 [0.95, 1.00]
Q-80B - rule	1.28 [1.10, 1.48]	0.99 [0.86, 1.12]	0.95 [0.77, 1.14]	1.80 [1.58, 2.04]	0.82 [0.71, 0.94]	0.37 [0.29, 0.48]	0.39 [0.30, 0.47]	0.55 [0.45, 0.68]	1.00 [1.00, 1.00]	0.99 [0.95, 1.00]

Supplementary Table 8 | MTB Patient Letters: Clinical Harm Rate (lower = better). CHR = 1 − recall; proportion of sections with major/critical errors missed by the LLM judge.

	CHR
G-120B - baseline	0.63 [0.52, 0.72]
G-120B - rule	0.67 [0.58, 0.75]
G-120B - demo	0.54 [0.42, 0.66]
M-24B - baseline	0.77 [0.69, 0.85]
M-24B - rule	0.73 [0.64, 0.81]
M-24B - demo	0.73 [0.63, 0.81]
MG-27B - baseline	0.77 [0.67, 0.84]
MG-27B - rule	0.87 [0.78, 0.93]
MG-27B - demo	0.86 [0.79, 0.92]
MULTIAGENT - multiagent	0.52 [0.42, 0.61]
Q-235B - baseline	0.20 [0.13, 0.29]
Q-235B - rule	0.10 [0.05, 0.18]
Q-235B - demo	0.35 [0.27, 0.45]
Q-235B-Think - baseline	0.45 [0.37, 0.55]
Q-235B-Think - rule	0.38 [0.29, 0.48]
Q-235B-Think - demo	0.50 [0.41, 0.60]
Q-80B - baseline	0.44 [0.34, 0.53]
Q-80B - rule	0.41 [0.32, 0.50]
Q-80B - demo	0.27 [0.19, 0.36]

Supplementary Table 9 | Discharge Summary Pairs: Accuracy on Clear-Majority Items by Criterion.

	clinical_utility	organized	completeness	efficiency	human_written
G-120B - baseline	0.62 [0.38, 0.87]	0.79 [0.50, 0.93]	0.61 [0.39, 0.83]	0.56 [0.31, 0.81]	0.42 [0.27, 0.61]
G-120B - rule	0.50 [0.25, 0.75]	0.71 [0.43, 0.93]	0.67 [0.44, 0.83]	0.75 [0.50, 0.94]	0.45 [0.27, 0.64]
G-120B - demo	0.69 [0.44, 0.88]	0.86 [0.57, 1.00]	0.67 [0.44, 0.83]	0.75 [0.50, 0.94]	0.76 [0.61, 0.91]
M-24B - baseline	0.50 [0.25, 0.75]	0.29 [0.07, 0.57]	0.39 [0.22, 0.61]	0.69 [0.44, 0.88]	0.70 [0.52, 0.85]
M-24B - rule	0.56 [0.31, 0.81]	0.50 [0.21, 0.79]	0.50 [0.28, 0.72]	0.69 [0.44, 0.88]	0.79 [0.64, 0.91]
M-24B - demo	0.50 [0.25, 0.75]	0.50 [0.29, 0.79]	0.39 [0.17, 0.67]	0.81 [0.56, 0.94]	0.70 [0.52, 0.85]
MG-27B - baseline	0.56 [0.31, 0.81]	0.71 [0.43, 0.93]	0.61 [0.39, 0.83]	0.38 [0.19, 0.62]	0.76 [0.61, 0.88]
MG-27B - rule	0.56 [0.31, 0.81]	0.71 [0.43, 0.93]	0.39 [0.17, 0.67]	0.50 [0.31, 0.75]	0.64 [0.48, 0.79]
MG-27B - demo	0.62 [0.38, 0.81]	0.79 [0.50, 0.93]	0.67 [0.44, 0.83]	0.62 [0.38, 0.88]	0.79 [0.64, 0.91]
MULTIAGENT - multiagent	0.44 [0.19, 0.69]	0.71 [0.45, 0.93]	0.33 [0.17, 0.56]	0.75 [0.50, 0.94]	0.42 [0.27, 0.61]
Q-235B - baseline	0.38 [0.19, 0.62]	0.71 [0.45, 0.93]	0.28 [0.11, 0.56]	0.81 [0.56, 0.94]	0.30 [0.18, 0.49]
Q-235B - rule	0.38 [0.19, 0.62]	0.43 [0.21, 0.71]	0.28 [0.11, 0.56]	0.62 [0.38, 0.81]	0.42 [0.27, 0.62]
Q-235B - demo	0.44 [0.19, 0.69]	0.71 [0.43, 0.93]	0.44 [0.22, 0.67]	0.81 [0.56, 0.94]	0.58 [0.42, 0.74]
Q-235B-Think - baseline	0.62 [0.38, 0.88]	0.57 [0.29, 0.79]	0.67 [0.39, 0.83]	0.56 [0.31, 0.81]	0.61 [0.42, 0.76]
Q-235B-Think - rule	0.62 [0.38, 0.81]	0.50 [0.21, 0.79]	0.61 [0.33, 0.83]	0.56 [0.31, 0.81]	0.64 [0.45, 0.79]
Q-235B-Think - demo	0.56 [0.31, 0.81]	0.57 [0.29, 0.79]	0.61 [0.33, 0.83]	0.62 [0.38, 0.82]	0.82 [0.67, 0.91]
Q-80B - baseline	0.44 [0.19, 0.69]	0.71 [0.43, 0.93]	0.33 [0.17, 0.56]	0.75 [0.50, 0.94]	0.70 [0.52, 0.85]
Q-80B - rule	0.50 [0.25, 0.75]	0.64 [0.36, 0.86]	0.33 [0.17, 0.56]	0.62 [0.38, 0.81]	0.70 [0.52, 0.85]
Q-80B - demo	0.56 [0.31, 0.81]	0.71 [0.43, 0.93]	0.39 [0.22, 0.61]	0.88 [0.62, 1.00]	0.64 [0.45, 0.79]

Complete Reliability Results

Supplementary Table 10 | Clinical Report Summaries: Gwet's AC2 (quadratic) by Criterion (higher = better). Human-human macro AC2 per subtype: radiology = 0.853, pathology = 0.868, surgery = 0.943.

	clinical_utility	accurate	thorough	useful	succinct	comprehensible	organized
G-120B - baseline	0.83 [0.78, 0.88]	0.89 [0.86, 0.92]	0.84 [0.80, 0.88]	0.87 [0.83, 0.92]	0.67 [0.58, 0.75]	0.88 [0.85, 0.90]	0.97 [0.96, 0.98]
G-120B - rule	0.73 [0.67, 0.79]	0.84 [0.81, 0.87]	0.83 [0.77, 0.88]	0.86 [0.80, 0.91]	0.67 [0.58, 0.75]	0.86 [0.83, 0.88]	0.90 [0.88, 0.91]
G-120B - demo	0.86 [0.82, 0.91]	0.90 [0.87, 0.92]	0.91 [0.88, 0.94]	0.90 [0.87, 0.94]	0.68 [0.59, 0.76]	0.88 [0.86, 0.91]	0.97 [0.96, 0.98]
M-24B - baseline	0.86 [0.81, 0.91]	0.85 [0.82, 0.88]	0.98 [0.97, 0.99]	0.75 [0.68, 0.81]	0.67 [0.58, 0.75]	0.85 [0.82, 0.88]	0.88 [0.87, 0.89]
M-24B - rule	0.91 [0.88, 0.95]	0.84 [0.82, 0.87]	0.98 [0.97, 0.99]	0.75 [0.69, 0.82]	0.78 [0.71, 0.85]	0.85 [0.82, 0.88]	0.89 [0.87, 0.90]
M-24B - demo	0.84 [0.78, 0.89]	0.90 [0.87, 0.93]	0.96 [0.94, 0.98]	0.80 [0.73, 0.86]	0.71 [0.63, 0.80]	0.89 [0.87, 0.92]	0.91 [0.89, 0.92]
MG-27B - baseline	0.73 [0.67, 0.78]	0.84 [0.82, 0.87]	0.96 [0.94, 0.98]	0.74 [0.68, 0.81]	0.67 [0.58, 0.75]	0.85 [0.82, 0.88]	0.89 [0.88, 0.91]
MG-27B - rule	0.78 [0.72, 0.84]	0.84 [0.82, 0.87]	0.98 [0.98, 0.99]	0.74 [0.68, 0.80]	0.67 [0.58, 0.75]	0.85 [0.82, 0.88]	0.91 [0.88, 0.93]
MG-27B - demo	0.73 [0.67, 0.78]	0.84 [0.82, 0.87]	0.96 [0.95, 0.98]	0.74 [0.68, 0.80]	0.67 [0.58, 0.75]	0.85 [0.82, 0.88]	0.89 [0.88, 0.91]
MULTIAGENT - multiagent	0.81 [0.76, 0.86]	0.84 [0.81, 0.87]	0.97 [0.96, 0.98]	0.79 [0.72, 0.85]	0.67 [0.58, 0.75]	0.85 [0.82, 0.88]	0.88 [0.87, 0.89]
Q-235B - baseline	0.81 [0.76, 0.86]	0.84 [0.81, 0.87]	0.96 [0.95, 0.98]	0.78 [0.72, 0.84]	0.67 [0.58, 0.75]	0.85 [0.82, 0.88]	0.88 [0.87, 0.89]
Q-235B - rule	0.90 [0.87, 0.93]	0.85 [0.82, 0.87]	0.88 [0.85, 0.91]	0.76 [0.69, 0.83]	0.67 [0.58, 0.76]	0.85 [0.82, 0.88]	0.88 [0.87, 0.89]
Q-235B - demo	0.74 [0.68, 0.80]	0.84 [0.81, 0.87]	0.99 [0.98, 0.99]	0.74 [0.68, 0.81]	0.67 [0.59, 0.76]	0.85 [0.82, 0.88]	0.88 [0.87, 0.89]
Q-235B-Think - baseline	-0.14 [-0.21, -0.07]	0.86 [0.84, 0.89]	0.93 [0.91, 0.95]	0.78 [0.73, 0.84]	0.67 [0.58, 0.75]	0.86 [0.83, 0.88]	0.88 [0.87, 0.89]
Q-235B-Think - rule	0.07 [-0.07, 0.21]	0.80 [0.76, 0.85]	0.37 [0.23, 0.50]	0.73 [0.67, 0.80]	0.66 [0.57, 0.75]	0.85 [0.83, 0.88]	0.88 [0.86, 0.89]
Q-235B-Think - demo	0.76 [0.68, 0.85]	0.87 [0.85, 0.90]	0.93 [0.91, 0.95]	0.80 [0.74, 0.85]	0.67 [0.58, 0.75]	0.86 [0.84, 0.88]	0.88 [0.87, 0.90]
Q-80B - baseline	0.73 [0.67, 0.79]	0.85 [0.82, 0.87]	0.97 [0.96, 0.98]	0.75 [0.68, 0.81]	0.67 [0.58, 0.75]	0.85 [0.82, 0.88]	0.88 [0.87, 0.89]
Q-80B - rule	0.84 [0.79, 0.89]	0.85 [0.82, 0.87]	0.95 [0.93, 0.96]	0.74 [0.68, 0.80]	0.67 [0.59, 0.76]	0.85 [0.82, 0.88]	0.89 [0.88, 0.90]
Q-80B - demo	0.73 [0.68, 0.78]	0.84 [0.82, 0.87]	0.97 [0.95, 0.98]	0.74 [0.68, 0.81]	0.67 [0.58, 0.75]	0.85 [0.82, 0.88]	0.88 [0.87, 0.89]

Supplementary Table 11 | Nursing Handover Notes: Gwet's AC2 by Criterion (higher = better)
Human-human macro AC2: 0.733 (combined); summary = 0.712, endpoints = 0.755.

	accurate	thorough	citation	abstraction	useful	succinct	comprehensible	organized	stig_original	stig_summary
G-120B - baseline	0.65 [0.55, 0.75]	0.62 [0.52, 0.73]	0.53 [0.38, 0.69]	0.61 [0.53, 0.69]	0.88 [0.84, 0.92]	0.94 [0.93, 0.96]	0.96 [0.95, 0.97]	0.91 [0.89, 0.94]	1.00 [1.00, 1.00]	0.99 [0.97, 1.00]
G-120B - demo	0.66 [0.55, 0.77]	0.63 [0.53, 0.73]	0.67 [0.54, 0.80]	0.69 [0.61, 0.77]	0.91 [0.88, 0.94]	0.94 [0.93, 0.96]	0.96 [0.95, 0.97]	0.91 [0.89, 0.94]	1.00 [1.00, 1.00]	0.99 [0.97, 1.00]
G-120B - rule	0.49 [0.37, 0.62]	0.20 [0.05, 0.35]	0.60 [0.49, 0.71]	0.55 [0.46, 0.64]	0.75 [0.68, 0.82]	0.96 [0.95, 0.97]	0.97 [0.95, 0.98]	0.92 [0.89, 0.94]	1.00 [1.00, 1.00]	0.99 [0.97, 1.00]
M-24B - baseline	0.54 [0.42, 0.67]	0.92 [0.89, 0.95]	0.51 [0.35, 0.66]	0.95 [0.93, 0.97]	0.87 [0.84, 0.91]	0.95 [0.93, 0.96]	0.97 [0.96, 0.98]	0.91 [0.89, 0.94]	0.95 [0.90, 0.99]	0.99 [0.97, 1.00]
M-24B - demo	0.53 [0.40, 0.66]	0.87 [0.82, 0.92]	0.52 [0.37, 0.68]	0.95 [0.93, 0.97]	0.87 [0.83, 0.91]	0.93 [0.91, 0.95]	0.97 [0.95, 0.98]	0.91 [0.88, 0.93]	1.00 [1.00, 1.00]	0.99 [0.97, 1.00]
M-24B - rule	0.53 [0.40, 0.66]	0.88 [0.84, 0.93]	0.51 [0.36, 0.67]	0.79 [0.74, 0.84]	0.90 [0.86, 0.93]	0.95 [0.93, 0.96]	0.95 [0.94, 0.96]	0.91 [0.89, 0.94]	0.98 [0.95, 1.00]	0.99 [0.97, 1.00]
MG-27B - baseline	0.54 [0.41, 0.66]	0.92 [0.87, 0.96]	0.54 [0.39, 0.69]	0.91 [0.88, 0.95]	0.86 [0.82, 0.91]	0.93 [0.92, 0.95]	0.97 [0.96, 0.98]	0.91 [0.89, 0.94]	0.98 [0.95, 1.00]	0.97 [0.93, 1.00]
MG-27B - demo	0.53 [0.40, 0.65]	0.92 [0.87, 0.96]	0.55 [0.40, 0.70]	0.90 [0.87, 0.94]	0.85 [0.81, 0.90]	0.95 [0.93, 0.96]	0.97 [0.96, 0.98]	0.91 [0.89, 0.94]	1.00 [1.00, 1.00]	0.99 [0.97, 1.00]
MG-27B - rule	0.53 [0.40, 0.65]	0.91 [0.87, 0.94]	0.63 [0.51, 0.75]	0.32 [0.14, 0.51]	0.86 [0.82, 0.90]	0.95 [0.93, 0.97]	0.97 [0.96, 0.98]	0.91 [0.89, 0.94]	1.00 [1.00, 1.00]	0.99 [0.97, 1.00]
MULTIAGENT - multiagent	0.54 [0.41, 0.67]	0.92 [0.89, 0.96]	0.51 [0.36, 0.66]	0.93 [0.91, 0.96]	0.87 [0.83, 0.91]	0.95 [0.94, 0.96]	0.97 [0.96, 0.98]	0.91 [0.89, 0.94]	0.96 [0.91, 1.00]	0.99 [0.97, 1.00]
Q-235B - baseline	0.54 [0.41, 0.67]	0.92 [0.89, 0.96]	0.51 [0.36, 0.66]	0.93 [0.91, 0.95]	0.87 [0.83, 0.91]	0.95 [0.94, 0.97]	0.97 [0.96, 0.98]	0.91 [0.89, 0.94]	0.70 [0.57, 0.83]	0.99 [0.97, 1.00]
Q-235B - demo	0.54 [0.41, 0.67]	0.91 [0.87, 0.95]	0.54 [0.37, 0.70]	0.94 [0.92, 0.96]	0.87 [0.83, 0.91]	0.95 [0.94, 0.97]	0.97 [0.96, 0.98]	0.91 [0.89, 0.94]	0.91 [0.85, 0.98]	0.99 [0.97, 1.00]
Q-235B - rule	0.45 [0.33, 0.57]	0.39 [0.26, 0.53]	0.56 [0.41, 0.71]	-0.25 [-0.44, -0.06]	0.91 [0.88, 0.94]	0.95 [0.94, 0.97]	0.97 [0.96, 0.98]	0.91 [0.89, 0.94]	0.99 [0.97, 1.00]	0.99 [0.97, 1.00]
Q-235B-Think - baseline	0.61 [0.49, 0.74]	0.70 [0.62, 0.78]	0.64 [0.52, 0.77]	0.89 [0.86, 0.93]	0.88 [0.84, 0.93]	0.95 [0.93, 0.96]	0.97 [0.95, 0.98]	0.92 [0.90, 0.95]	0.98 [0.95, 1.00]	0.99 [0.97, 1.00]
Q-235B-Think - demo	0.68 [0.57, 0.78]	0.59 [0.47, 0.70]	0.71 [0.62, 0.81]	0.71 [0.63, 0.79]	0.81 [0.75, 0.87]	0.95 [0.94, 0.96]	0.96 [0.95, 0.98]	0.93 [0.91, 0.95]	1.00 [1.00, 1.00]	0.99 [0.97, 1.00]
Q-235B-Think - rule	0.58 [0.48, 0.67]	0.21 [0.09, 0.34]	0.58 [0.48, 0.68]	-0.21 [-0.38, -0.03]	0.87 [0.83, 0.92]	0.95 [0.93, 0.97]	0.97 [0.95, 0.98]	0.91 [0.89, 0.93]	1.00 [1.00, 1.00]	0.99 [0.97, 1.00]
Q-80B - baseline	0.53 [0.40, 0.66]	0.89 [0.84, 0.94]	0.51 [0.35, 0.66]	0.92 [0.89, 0.94]	0.86 [0.82, 0.90]	0.95 [0.94, 0.97]	0.96 [0.95, 0.98]	0.91 [0.89, 0.94]	0.96 [0.91, 1.00]	0.99 [0.97, 1.00]
Q-80B - demo	0.53 [0.41, 0.66]	0.86 [0.81, 0.91]	0.51 [0.35, 0.66]	0.94 [0.92, 0.96]	0.86 [0.82, 0.90]	0.95 [0.93, 0.96]	0.96 [0.95, 0.98]	0.91 [0.89, 0.94]	0.93 [0.88, 0.99]	0.99 [0.97, 1.00]
Q-80B - rule	0.54 [0.41, 0.67]	0.82 [0.75, 0.89]	0.63 [0.51, 0.75]	0.25 [0.09, 0.41]	0.87 [0.83, 0.91]	0.95 [0.94, 0.97]	0.95 [0.94, 0.97]	0.92 [0.89, 0.94]	1.00 [1.00, 1.00]	0.99 [0.97, 1.00]

Supplementary Table 12 | MTB Patient Letters: Gwet's AC1 (pass/fail binary). Human-human AC1: not reported (single annotation per section).

	CHR
G-120B - baseline	0.87 [0.84, 0.90]
G-120B - rule	0.85 [0.82, 0.88]
G-120B - demo	0.86 [0.82, 0.90]
M-24B - baseline	0.86 [0.83, 0.88]
M-24B - rule	0.88 [0.86, 0.91]
M-24B - demo	0.84 [0.81, 0.87]
MG-27B - baseline	0.81 [0.77, 0.84]
MG-27B - rule	0.85 [0.82, 0.88]
MG-27B - demo	0.82 [0.79, 0.85]
MULTIAGENT - multiagent	0.85 [0.82, 0.88]
Q-235B - baseline	0.70 [0.65, 0.74]
Q-235B - rule	0.52 [0.46, 0.58]
Q-235B - demo	0.77 [0.73, 0.81]
Q-235B-Think - baseline	0.82 [0.78, 0.85]
Q-235B-Think - rule	0.80 [0.76, 0.83]
Q-235B-Think - demo	0.84 [0.81, 0.87]
Q-80B - baseline	0.78 [0.74, 0.82]
Q-80B - rule	0.81 [0.78, 0.85]
Q-80B - demo	0.73 [0.69, 0.78]

Supplementary Table 13 | Discharge Summary Pairs: Gwet's AC1 by Criterion (higher = better). Human-human macro AC1 (pairwise): 0.567 [0.526,0.608].

	clinical_utility	organized	completeness	efficiency	human_written
G-120B - baseline	0.29 [0.05, 0.54]	0.49 [0.26, 0.73]	0.21 [-0.03, 0.46]	0.30 [0.05, 0.55]	0.28 [0.04, 0.52]
G-120B - rule	0.20 [-0.04, 0.44]	0.37 [0.12, 0.62]	0.21 [-0.04, 0.46]	0.41 [0.17, 0.66]	0.32 [0.08, 0.56]
G-120B - demo	0.30 [0.06, 0.55]	0.54 [0.31, 0.77]	0.34 [0.09, 0.59]	0.45 [0.22, 0.69]	0.75 [0.57, 0.92]
M-24B - baseline	0.31 [0.07, 0.56]	0.17 [-0.08, 0.42]	0.07 [-0.17, 0.31]	0.41 [0.17, 0.66]	0.67 [0.48, 0.87]
M-24B - rule	0.31 [0.07, 0.56]	0.33 [0.08, 0.58]	0.16 [-0.09, 0.41]	0.45 [0.22, 0.69]	0.78 [0.62, 0.94]
M-24B - demo	0.26 [0.01, 0.51]	0.29 [0.04, 0.54]	0.19 [-0.06, 0.44]	0.58 [0.36, 0.80]	0.67 [0.48, 0.87]
MG-27B - baseline	0.27 [0.02, 0.52]	0.56 [0.34, 0.78]	0.21 [-0.03, 0.46]	0.26 [0.01, 0.51]	0.75 [0.57, 0.92]
MG-27B - rule	0.27 [0.02, 0.52]	0.44 [0.20, 0.68]	0.03 [-0.20, 0.26]	0.35 [0.10, 0.59]	0.60 [0.39, 0.81]
MG-27B - demo	0.30 [0.05, 0.56]	0.52 [0.29, 0.75]	0.39 [0.14, 0.63]	0.51 [0.27, 0.74]	0.78 [0.62, 0.94]
MULTIAGENT - multiagent	0.19 [-0.06, 0.44]	0.51 [0.28, 0.74]	-0.01 [-0.23, 0.22]	0.45 [0.22, 0.69]	0.28 [0.04, 0.52]
Q-235B - baseline	0.13 [-0.11, 0.37]	0.55 [0.32, 0.77]	-0.05 [-0.27, 0.16]	0.61 [0.40, 0.83]	0.11 [-0.11, 0.34]
Q-235B - rule	0.09 [-0.15, 0.32]	0.17 [-0.08, 0.42]	-0.06 [-0.27, 0.16]	0.49 [0.25, 0.73]	0.32 [0.08, 0.56]
Q-235B - demo	0.09 [-0.15, 0.34]	0.42 [0.18, 0.67]	0.08 [-0.16, 0.32]	0.61 [0.40, 0.83]	0.52 [0.30, 0.75]
Q-235B-Think - baseline	0.39 [0.14, 0.63]	0.30 [0.05, 0.55]	0.37 [0.12, 0.62]	0.34 [0.09, 0.59]	0.56 [0.34, 0.78]
Q-235B-Think - rule	0.38 [0.13, 0.62]	0.28 [0.03, 0.54]	0.36 [0.11, 0.61]	0.34 [0.09, 0.59]	0.60 [0.39, 0.81]
Q-235B-Think - demo	0.30 [0.06, 0.55]	0.33 [0.08, 0.58]	0.29 [0.04, 0.55]	0.39 [0.14, 0.63]	0.81 [0.67, 0.96]
Q-80B - baseline	0.29 [0.05, 0.54]	0.50 [0.27, 0.73]	-0.01 [-0.24, 0.21]	0.59 [0.37, 0.81]	0.67 [0.48, 0.87]
Q-80B - rule	0.33 [0.09, 0.58]	0.50 [0.27, 0.73]	-0.01 [-0.24, 0.21]	0.50 [0.26, 0.73]	0.64 [0.44, 0.84]
Q-80B - demo	0.42 [0.18, 0.66]	0.50 [0.27, 0.73]	0.08 [-0.16, 0.32]	0.59 [0.37, 0.81]	0.60 [0.39, 0.81]
